# Construction of an immune‐related prognostic model and potential drugs screening for esophageal cancer based on bioinformatics analyses and network pharmacology

**DOI:** 10.1002/iid3.1266

**Published:** 2024-05-28

**Authors:** Pengju Qi, Bo Qi, Chengwei Gu, Shuhua Huo, Xinchen Dang, Yuzhen Liu, Baosheng Zhao

**Affiliations:** ^1^ Department of Thoracic Surgery The First Affiliated Hospital of Xinxiang Medical University Weihui Henan China; ^2^ Esophageal Cancer Institute of Xinxiang Medical University Weihui Henan China; ^3^ Life Science Research Center The First Affiliated Hospital of Xinxiang Medical University Weihui Henan China

**Keywords:** esophageal cancer, immune‐related gene, long noncoding RNA, network pharmacology, perphenazine, prognostic model

## Abstract

**Background:**

Esophageal cancer (ESCA) is a highly invasive malignant tumor with poor prognosis. This study aimed to discover a generalized and high‐sensitivity immune prognostic signature that could stratify ESCA patients and predict their overall survival, and to discover potential therapeutic drugs by the connectivity map.

**Methods:**

The key gene modules significantly related to clinical traits (survival time and state) of ESCA patients were selected by weighted gene coexpression network analysis (WCGNA), then the univariate and least absolute shrinkage and selection operator (LASSO) Cox regression analyses were used to construct a 15‐immune‐related gene prognostic signature.

**Results:**

The immune‐related risk model was related to clinical and pathologic factors and remained an effective independent prognostic factor. Enrichment analyses revealed that the differentially expressed genes (DEGs) of the high‐ and low‐risk groups were associated with tumor cell proliferation and immune mechanisms. Based on the gathered data, a small molecule drug named perphenazine (PPZ) was elected. The pharmacological analysis indicates that PPZ could help in adjuvant therapy of ESCA through regulation of metabolic process and cellular proliferation, enhancement of immunologic functions, and inhibition of inflammatory reactions. Furthermore, molecular docking was performed to explore and verify the PPZ‐core target interactions.

**Conclusion:**

We succeed in structuring the immune‐related prognostic model, which could be used to distinguish and predict patients' survival outcome, and screening a small molecule drug named PPZ. Prospective studies also are needed to further validate its analytical accuracy for estimating prognoses and confirm the potential use of PPZ for treating ESCA.

## 1 INTRODUCTION

The latest global cancer statistics in 2020 show that esophageal cancer (ESCA) ranked seventh and sixth in the incidence and mortality of malignant tumors. Although advances in diagnosis and treatment, the majority of patients with ESCA are still in middle or advanced stages when admitted to hospital for examination and the prognosis is poor, as illustrated by 5‐year survival rate of about 15%.^1^ Discovering promising diagnostic biomarkers and novel therapeutic targets as well as understanding the occurrence and development of ESCA at the molecular level is not only beneficial to clinical decision‐making, but also crucial to early disease diagnosis and treatment strategies.^2^, ^3^

Compared with WHO (World Health Organization) classification, molecular genetics can better explain the occurrence and development of tumors from root causes.^4^ The processing of RNA is systematically altered in cancer, which proves that RNA plays an important role in tumorigenesis, growth, and progression.^5^ With the development of genomics, a large number of studies have shown that the immune‐related genes (IRGs) may be related to cancer: Based on nine IRGs, Qu et al.^6^ identified and verified an individualized prognostic signature of bladder cancer, Zhang et al.^7^ found a novel IRG signature for risk stratification and prognosis of survival in lower‐grade glioma. Not only that, immune disorders are considered as promoting factors during tumorigenesis and cancer progression.^8^ On the other hand, the continuous development of transcriptomics has led people to further understand long noncoding RNAs (lncRNAs). Among its many functions, lncRNAs not only control gene expression in terms of epigenetic regulation, transcription progress, posttranscriptional modification, but they are also widely involved in physiological or pathological processes such as cell differentiation, cell multiplication and apoptosis, genomic imprinting, transcriptional activation, and inhibition.^9^ Additionally, in the cancer immunity response and the tumor microenvironment, lncRNAs have multiple functions.^10^, ^11^, ^12^ There is a significant amount of evidence supporting lncRNAs as possible diagnostic biomarkers and therapeutic targets,^13^ thereby lncRNAs cannot simply be regarded as the “noise” of genomic transcription. Considering the roles of mRNA and lncRNA in cancer biology, the prognostic signature that combines these two factors has a superior effect.^14^, ^15^, ^16^, ^17^ Advancement in bioinformatics introduces a wide spectrum of disease prediction and investigation of molecular mechanisms,^18^ allowing further research into the biological significance of immune‐related genes and lncRNAs in the development of disease.

The pathogenesis and resistance mechanism of many diseases are emergence, complexity, and robustness in human body system. Traditional high selectivity single‐target drugs have difficulty curing these complex diseases, such as malignant tumors, resulting in far lower than expected clinical effects. Network pharmacology is based on the drug molecule‐target gene‐disease network analytics, which can predict and reveal the active mechanism of drugs more systematically,^19^ even can discover new drug‐target combinations through biocomputing by integrating existing knowledge to clarify unknown mechanisms, allowing speculation of new pharmaceutical indications and implementation of rationally designed drug repositioning.^15^

Although many studies have investigated the relationship between IRGs and the prognosis of patients, few have generated a prognostic signature of IRGs in ESCA based on expression profile data. Because increasing numbers of lncRNAs are found to play a significant role in regulating mRNA stability, translation‐activity, and signal transduction pathway,^20^ we combined IRGs and their corresponding lncRNAs constructing a predictive risk model in ESCA for the first time, and the model established in this study could improve prognostic value via integrated bioinformatics approaches. Based upon the cMAP database, we screened perphenazine (PPZ), a small molecule drug suppressing the expression of high‐risk genes in our model. Network pharmacology approaches were used to explain the pharmacological mechanism more scientifically to ulteriorly lay a foundation for exploring the antitumor effects of PPZ.

## 2 MATERIALS AND METHODS

Materials and methods used in this study were provided in the Supporting Information.

## 3 RESULTS

### 3.1 Screening of IRGs related to ESCA prognosis

**Figure 1:**
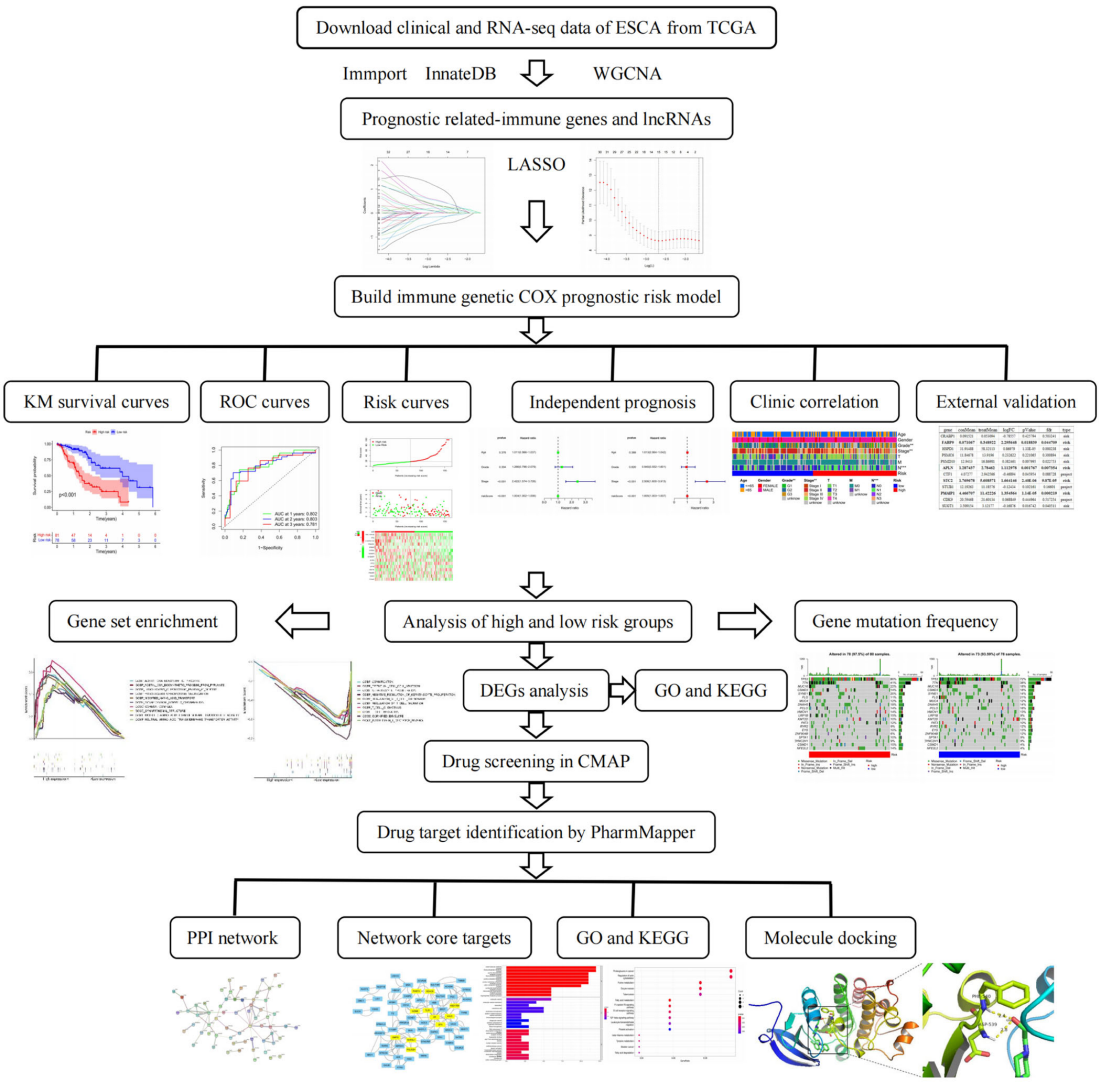
Flow diagram of this study. CMAP, connectivity map; DEGs, differentially expressed genes; ESCA, esophageal cancer; GO, gene ontology; KEGG, Kyoto Encyclopedia of Genes and Genomes; KM, Kaplan–Meier; LASSO, least absolute shrinkage and selection operator; lncRNAs, long noncoding RNAs; PPI, protein–protein interaction; ROC, receiver operating characteristic; TCGA, The Cancer Genome Atlas; WGCNA, weighted gene coexpression network analysis.

Figure 1 depicts a flowchart of the technical strategy used in this study. A total of 1976 IRGs were expressed in the samples of retrieved data sets, we then explored the relationship between coexpressed module and clinical phenotype by WGCNA (Supporting Information S1: Figure S1). The topological structure analysis of soft threshold parameters and hierarchical clustering tree based on the difference of adjacent values were shown in Supporting Information S1 (Figure S2). When we selected the prognosis‐related modules, the common problem, “curse‐of‐dimensionality” (small sample size combined with a very large number of genes) was taken into consideration. In comparison with other clinical phenotypes, we factored in this problem when narrowing the high dimension; thus, more attention was paid to survival time (futime) and status (fustat) that most closely related to prognosis. Subsequently, only the green and turquoise modules were significantly related to both the phenotypes at the same time (The red module was only negatively related to fustat), and the numbers of genes were 171 (green) and 510 (turquoise), respectively. The expression quantity of these 681 IRGs was extracted from the two‐dimensional matrix of IRGs and used for subsequent analysis.

### 3.2 Construction of the ESCA prognostic signature

**Figure 2:**
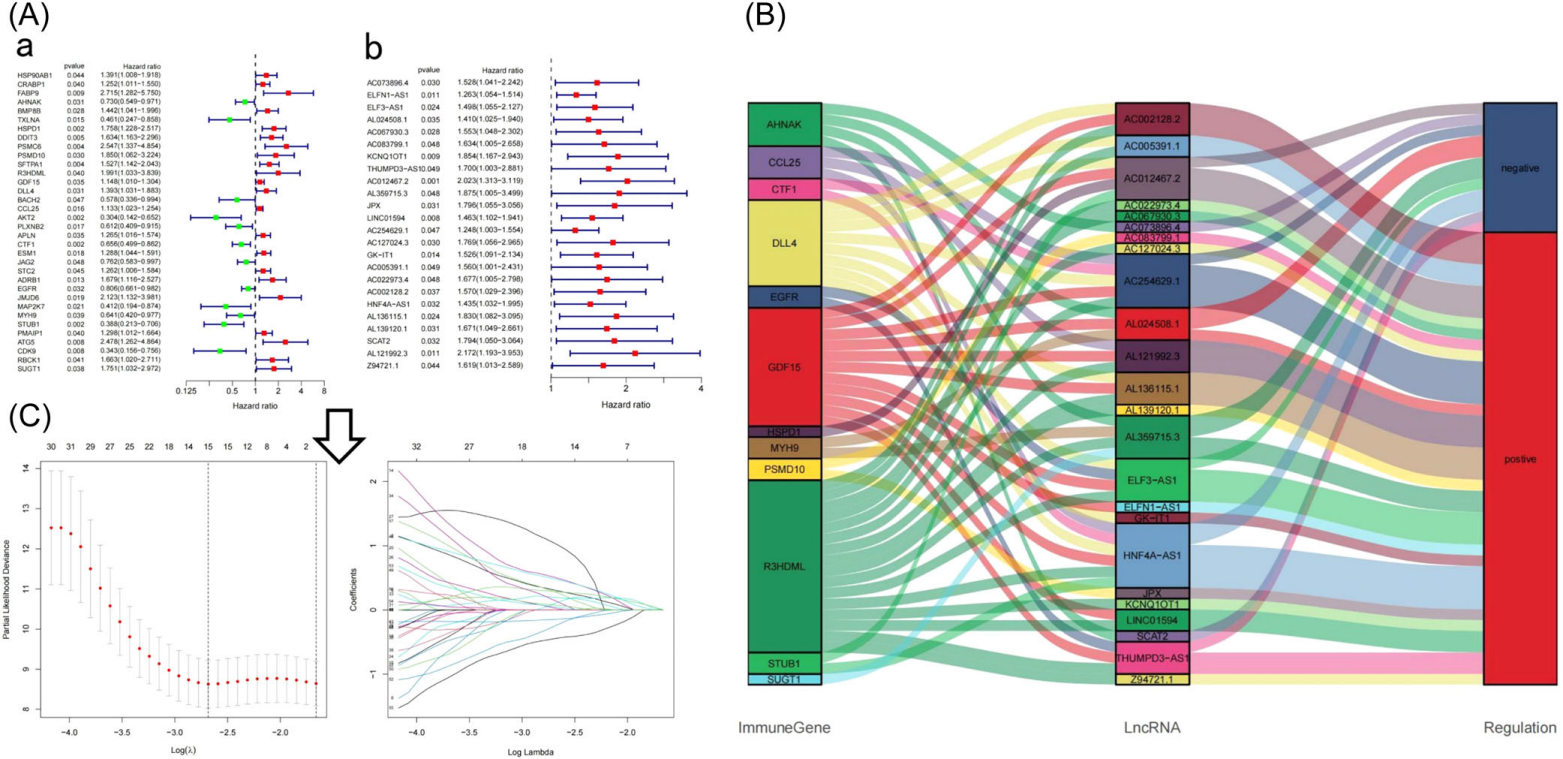
Construction of the immune‐related risk model. (A) Univariate Cox analysis identified survival‐related 34 mRNAs and 24 lncRNAs. *p* < .05 indicates a significant correlation between genes and prognosis, hazard ratio (HR) value > 1 means that the gene is a risk gene, and HR < 1 means a protective gene. (B) The best criteria to build the model based on LASSO regression. (C) Sankey diagram showing the coexpression relationship between the mRNAs and lncRNAs. LASSO, least absolute shrinkage and selection operator; lncRNAs, long noncoding RNAs; mRNA, messenger RNA.

The prognostic values of the 681 IRGs were analyzed by using univariate Cox regression analysis, 34 IRGs were found to be significantly linked to patient survival (Figure 2A‐a). The corresponding lncRNAs of the 34 IRGs were next selected according to the coexpression relationship of mRNA and lncRNA, and their prognostic values were also evaluated in the same way (Figure 2A‐b). All lncRNAs in this study were risky, Figure 2B showed the coexpression relationship. By LASSO analysis, we got 15 IRGs for structuring the immune‐related prognostic model, which were significantly associated with prognosis of ESCA patients (Figure 2C), the immune‐related risk score (IRS) formula was shown in the Supporting Information S1: Supplementary Results.

### 3.3 Validation of the ESCA prognostic signature

**Figure 3:**
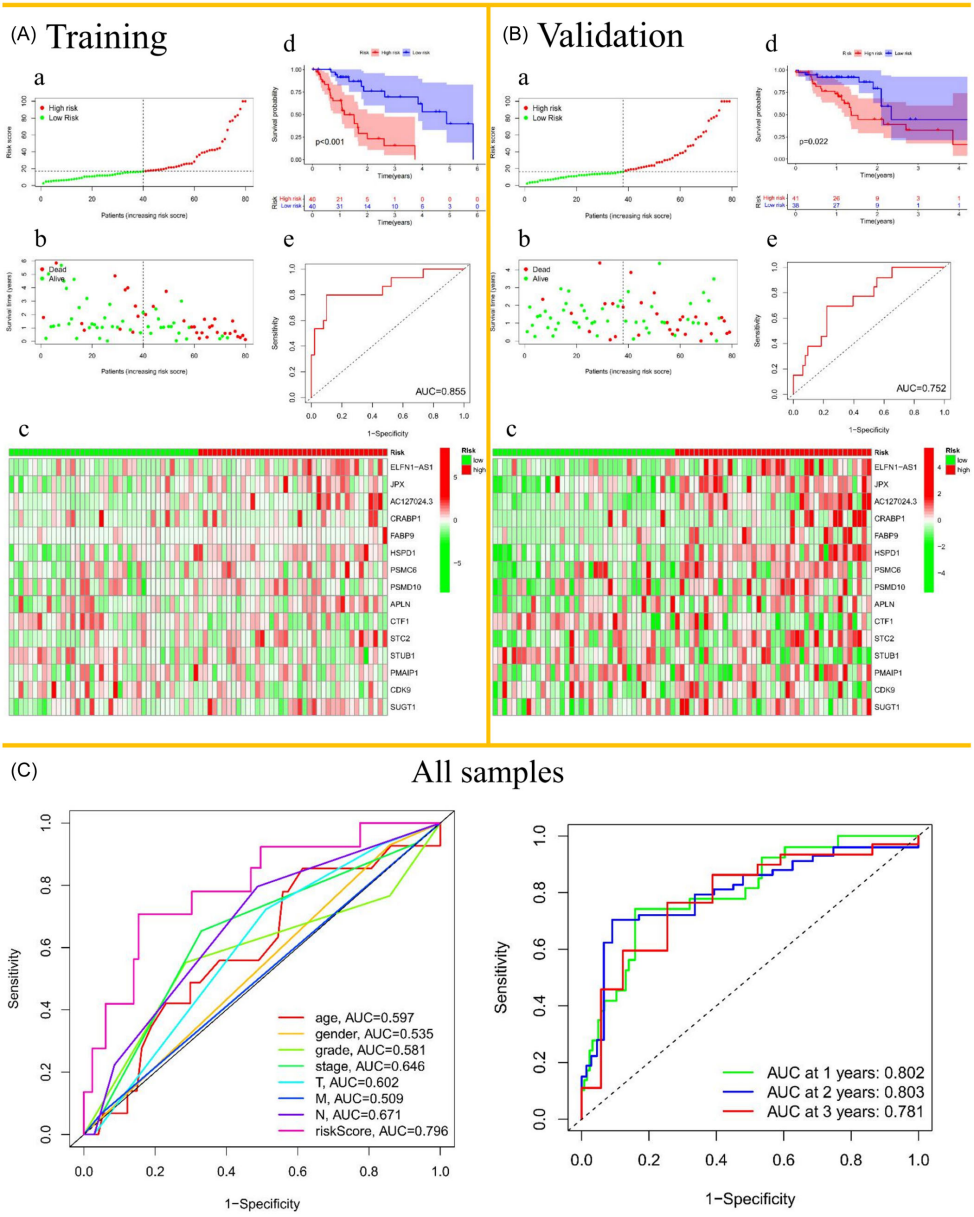
Verification between the immune‐related risk model. (A) and (B‐a to c). The distributions of risk scores (a), survival times and status (b), and the heatmaps of gene expression levels (c) in the training set and validation set. The black dotted lines represent the median risk score cut‐off dividing patients into high‐ and low‐risk groups. The red dots represent the ESCA patients in high‐risk group, and the green dots represent the low‐risk. (A) and (B‐d) Kaplan–Meier survival analyses of the ESCA patients. (A) and (B‐e) ROC curves of the training set and validation set. (C) Multi‐index ROC curves of age, gender, grade, stage, TNM, and risk score. Time‐dependent ROC curves for 1–3 years. AUC, area under the receiver operating characteristic curve; ESCA, esophageal cancer; ROC, receiver operating characteristic.

To assess the robustness of the prognostic model, we established training and validation sets and performed double validation in the overall set (Supporting Information S1: Figure S3). These ESCA patients in both training and validation sets were divided into high‐ and low‐risk groups, according to the median of IRS (Figure 3A,B‐a). After, it can be seen that as the IRS increases, more and more patients die, that is, the higher the IRS, the greater the number of deaths. Conversely, the lower the IRS, the longer the survival time of the ESCA patients. The distribution of survival status and survival time were shown in Figure 3A,B‐b. As the IRS decreases, the expression levels of risk genes also decreases, and vice versa. Expression patterns of the prognostic signature in the high‐ and low‐risk groups were shown in a heat map (Figure 3A,B‐c). The Kaplan Meier curves showed significant differences in overall survival (OS) between the high‐ and low‐risk groups whether in the training set or validation set. With the extension of survival time, the survival rate of the high‐risk group became lower, resulting in a poor prognostic effect (Figure 3A,B‐d). As for receiver operating characteristic (ROC) curves, the area under the receiver operating characteristic curves (AUCs) of the risk model in the training set and validation set were 0.855 and 0.752, respectively (Figure 3A,B‐e), strongly indicating that the risk model we established may have a good predictability for patient outcome. Furthermore, we drew the multiple indexes and points‐in‐time ROC curves of all patients and found that the AUC = 0.796 of the risk model was not only higher than other clinical phenotypes (age = 0.597, gender = 0.535, grade = 0.581, stage = 0.646, *T* = 0.602, *M* = 0.509, and *N* = 0.671) but also did not change with time (AUC for 1, 2, and 3 years were 0.802, 0.803, and 0.781, respectively) (Figure 3C). The prognostic model based on 15 IRGs was identified as an independent prognostic indicator by univariate and multivariate Cox analyses (Supporting Information S1: Figure S4).

**Figure 4:**
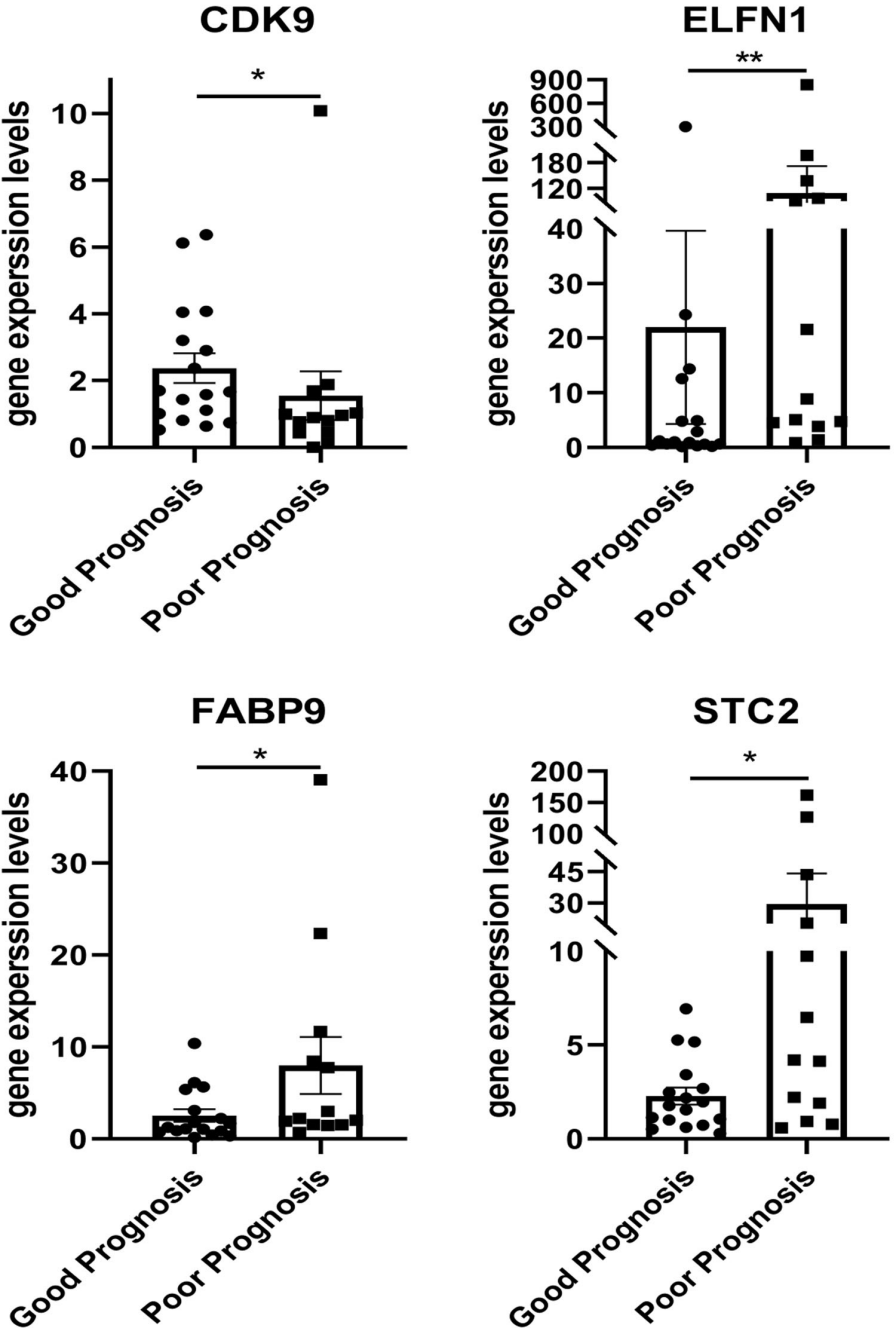
Quantitative polymerase chain reaction verification of *CDK9, ELNF1, FABP9*, and *STC2*.

There was a non‐negligible batch effect that cannot be eliminated between data from different platforms. Second, differentially expressed genes (DEGs) are not recommended for WGCNA, so we chose to select prognostic module genes to narrow the high dimension, rather than using DEGs in tumor and adjacent tissues for filtering. Based on the above reasons, we selected the tumor tissues of 30 patients whose prognoses had significant differences from our esophageal cancer sample database and extracted RNA for verify the stability of the prognostic model (clinical information of the 30 ESCA patients is shown in Supporting Information S1 (Table S1). Esophageal cancer patients whose survival time was more than or equal to 5 years were classified as good prognosis group and was less than 1 year were considered as poor prognosis group. Sequentially, the expression levels of the four model genes (*ELFN1‐AS1, FABP9, STC2*, and *CDK9*) that had high coefficient was detected in the tissues of patients with different prognosis to determine their potentials as prognostic markers. We found that ELFN1, FABP9, and STC2 of which model coefficient were 0.3066, 1.0331, and 0.4807, respectively, were highly expressed in the poor prognosis group, while the protective gene CDK9 (model coefficient was −0.445) was higher in the good prognosis group (Figure 4). The specific results of model comparison were shown in Supporting Information S1 (Figure S5). Collectively, these results indicate that our IRGs screened out in this study have a high reliability for constructing the risk model.

### 3.4 Analysis of the clinical correlation

**Figure 5:**
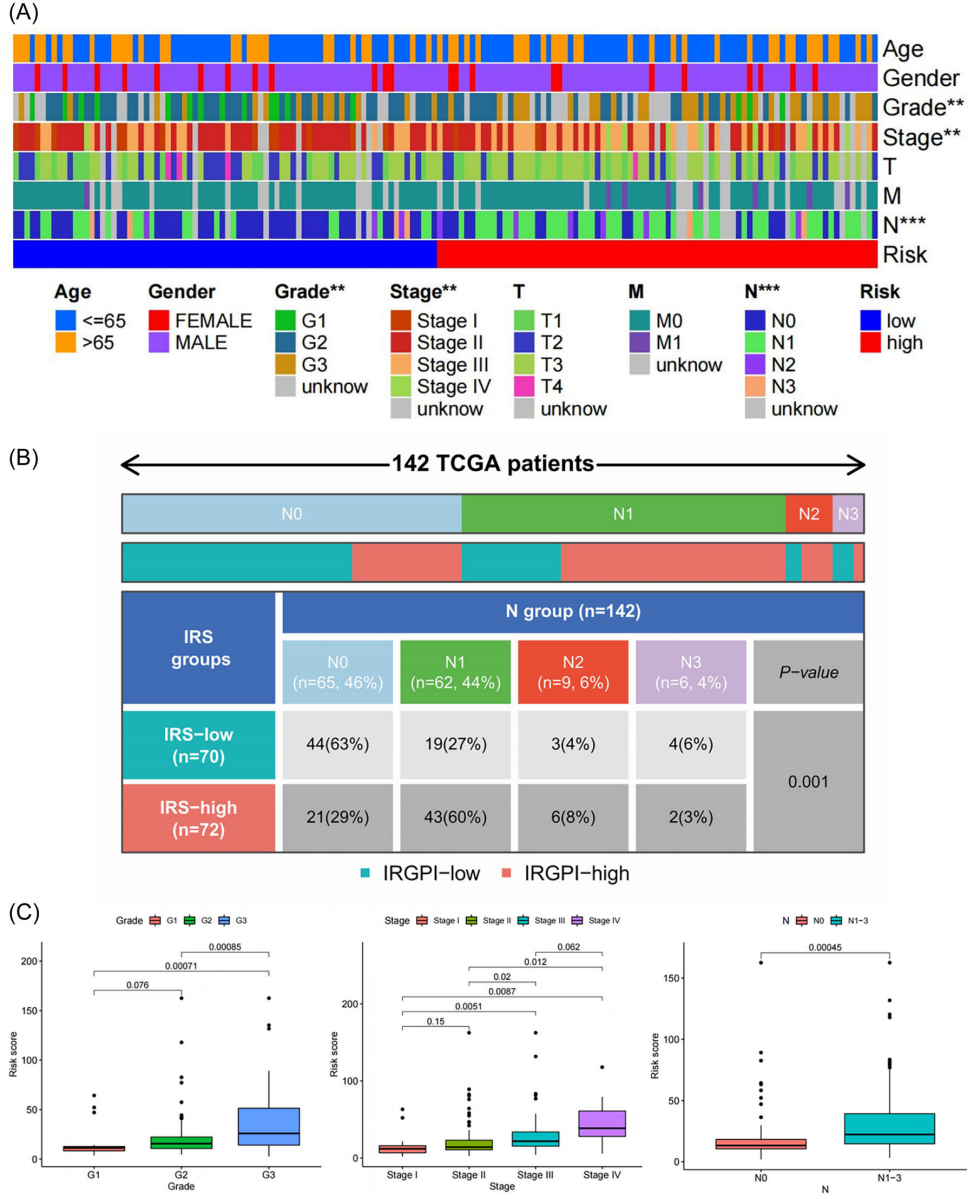
The heatmap of the clinical phenotypes (A) and distribution characteristic of pathological stage‐N (B) in the high‐ and low‐risk groups. (C) Differences in risk scores among different clinical features. IRGPI, immune‐related gene prognostic index; IRS, immune‐related risk score; TCGA, The Cancer Genome Atlas.

To evaluate the clinical prognostic value of the 15‐IRG signatures in patients with ESCA, we chose *χ* ^2^ test to analyze the relationship between the risk model and the clinical features of ESCA including age, gender, grade, stage, pathological stage‐T, ‐N, ‐M, alcohol, and tobacco (T: primary tumor size and scope, N: regional lymph node metastasis, M: distance metastases, Supporting Information S1: Table S2). In the training set, there were significant differences in grade and pathological stage‐N between high‐ and low‐risk groups, stage and pathological stage‐N were found existing significant differences in the validation set (Supporting Information S1: Table S3). The grade, stage, and pathological stage‐N of all patients displayed significant differences between high‐ and low‐risk groups (Supporting Information S1: Table S2, Figure 5A). Moreover, we found that pathological stage‐N was significantly related to the risk model either in the training set or in the validation set. Therefore, we further analyzed the specific distribution of pathological stage‐N (Figure 5B). The main difference was that the number of patients with pathological stage‐N1 in the high‐risk group was much greater than that of the low‐risk group, while patients with stage‐N0 were mainly concentrated in the low‐risk group. From another perspective, we further investigated the IRS of different clinicopathological characteristics by boxplot and found that there also were significant differences in grade, stage, and pathological stage‐N (Figure 5C). Patients with G3, stages III–IV, and N1‐3 were at great risk, which was consistent with the results of Figure 5A, indicating that clinical outcomes of adverse events such as tumor progression could be foreseen by evaluating the risk score using this model. Collectively, these results imply that the prognostic model developed in this study had great clinical relevance and could yield more accurate prediction for the prognosis of ESCA patients compared with these clinical features.

### 3.5 Analysis of tumor microenvironment (TME) and immune cell infiltration (ICI)

To further explore the underlying biological mechanisms resulting in differential prognosis between the high‐ and low‐risk groups, we calculated the stromal (measuring the presence of tumor‐associated stroma), immune (representing the infiltration level of the immune cells), and ESTIMATE (combining stromal and immune score to infer tumor purity comprehensively) scores based on the TCGA expression profiles. The results demonstrated that these three scores in the high and low‐risk groups were similar (Supporting Information S1: Figure S6A), suggesting that the tumor purity in the two groups was consistent. Herein, we analyzed the infiltration statuses of tumor‐infiltrating immunocytes (TIICs) to explore the reason why the tumor purity was similar, but the survival prognosis was very different. Supporting Information S1 (Figure S6B) showed that the content of B cell memory was generally lower in the high‐risk group, which might be an important reason for the poor prognosis.

### 3.6 Gene set enrichment analysis

**Figure 6:**
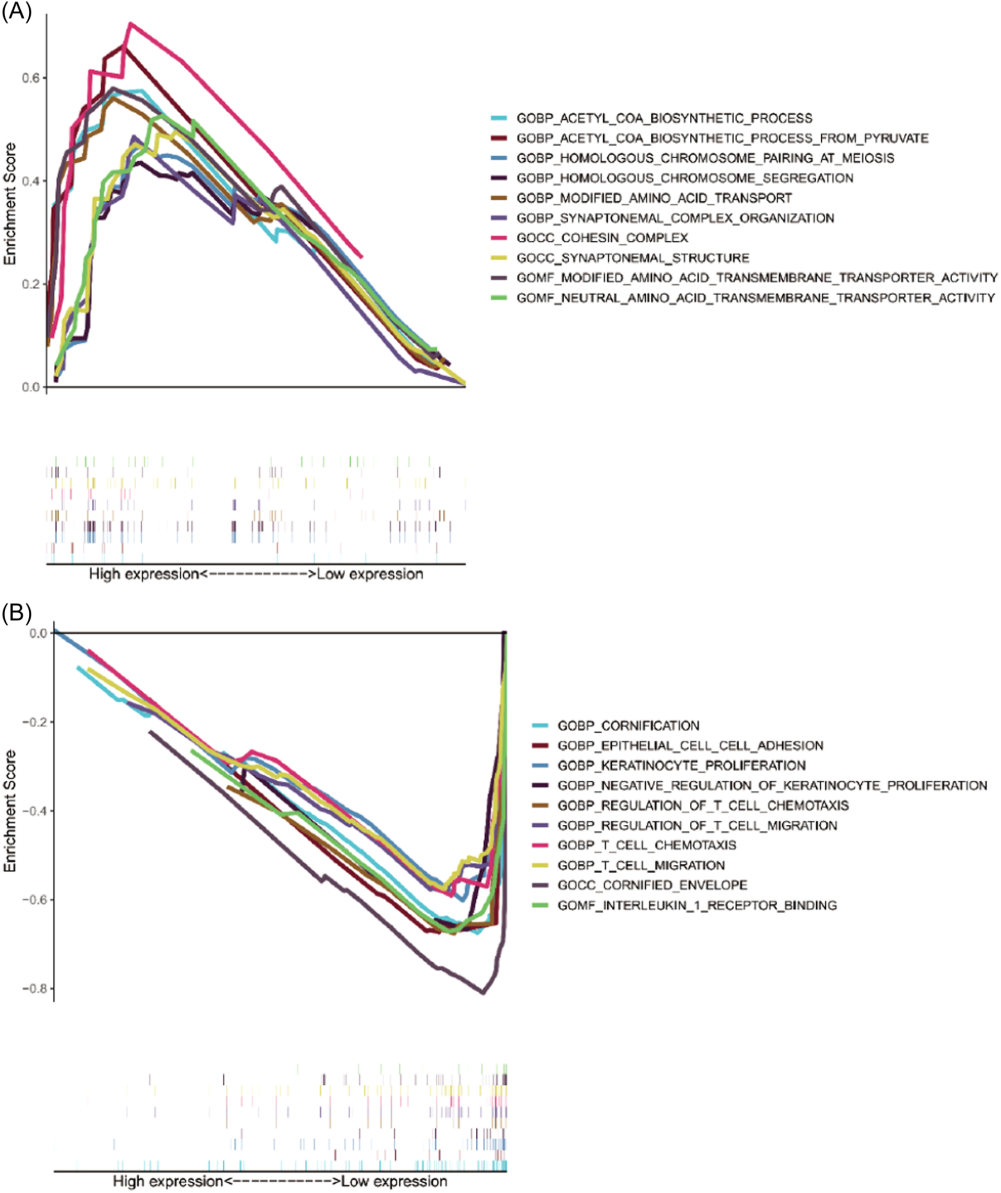
GO analysis of Gene Set Enrichment Analysis (GSEA) in the high‐ (A) and low‐risk (B) groups. GO, gene ontology.

To elucidate functional enrichment between the two groups of ESCA patients, Gene Set Enrichment Analysis (GSEA) was performed. The major gene ontology (GO) terms enriched were shown in Figure 6. The key functions related to metabolization and proliferation, such as the acetyl‐CoA biosynthetic process, modified amino acid transport, homologous chromosome pairing at meiosis, and synaptonemal complex organization, were significantly enriched in the high‐risk group (Figure 6A), and the major functions enriched in the low‐risk group were related to cornification and immunization, including keratinocyte proliferation, epithelial cell‐cell adhesion, regulation of T‐cell chemotaxis, T‐cell migration (Figure 6B).

### 3.7 Gene mutation analysis

Waterfall plot represented the mutational landscape of the top 20 most frequently mutated genes in the high‐ (Supporting Information S1: Figure S7A) and low‐risk (Supporting Information S1: Figure S7B) groups, with different colors signifying different mutation types. The results revealed that the most common mutation in ESCA patients was the missense mutation, which was mainly caused by SNPs (single nucleotide polymorphisms). *TP53* had the highest mutation frequency (mutated in 80% and 72% of the high‐ and low‐risk groups, respectively), followed by *TTN, MUC16*, and so on. On the other hand, the gene mutation spectrums showed that the two groups had similar mutation frequency (Supporting Information S1: Figure S8).

### 3.8 GO and Kyoto Encyclopedia of Genes and Genomes (KEGG) enrichment analysis of DEGs

**Figure 7:**
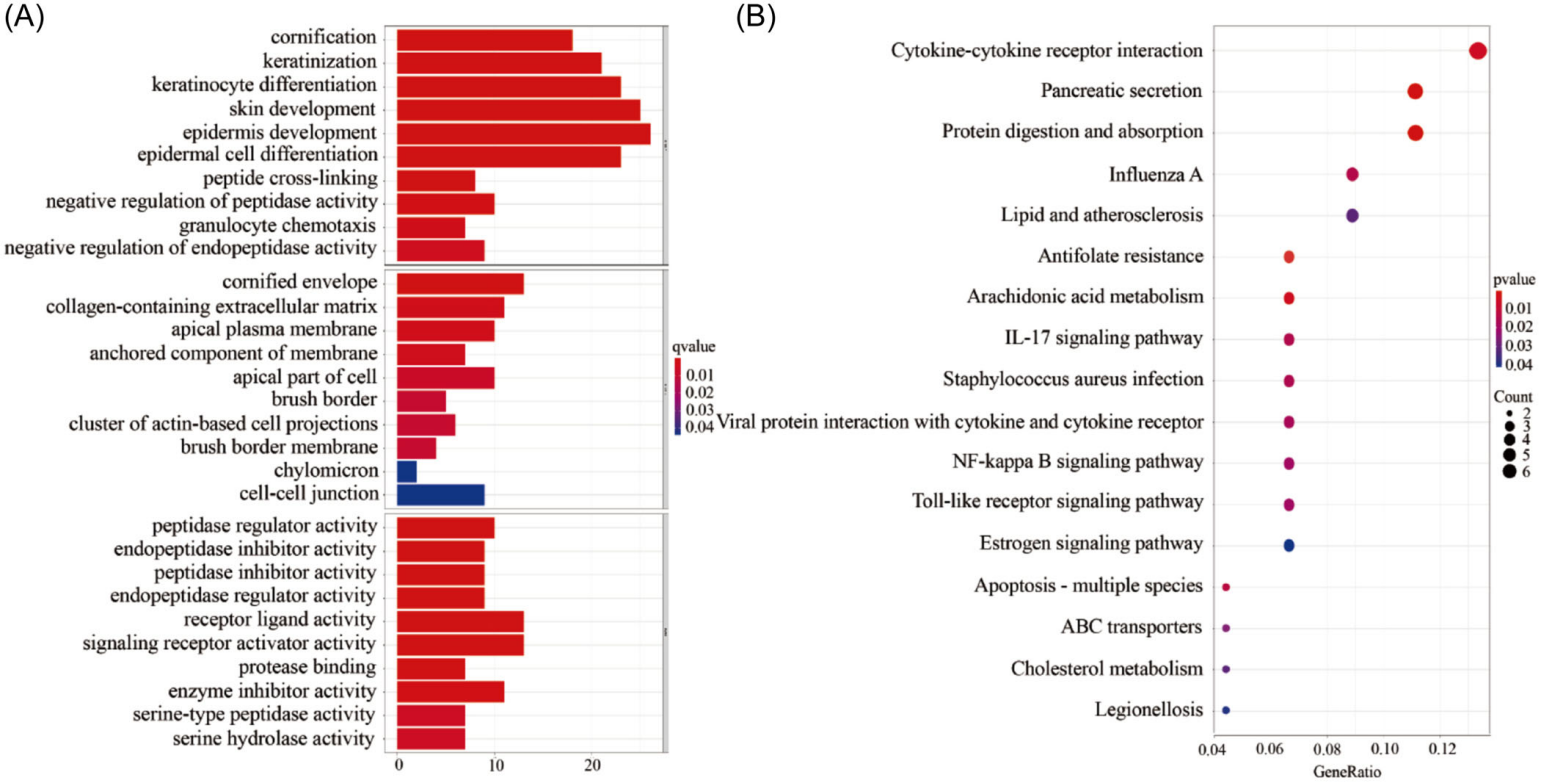
The enrichment analysis of DEGs. (A) GO functional enrichment analysis. (B) KEGG pathway enrichment analysis. Circle color indicates *P*. Circle size represents the gene number enriched in the pathway. DEGs, differentially expressed genes; GO, gene ontology; KEGG, Kyoto Encyclopedia of Genes and Genomes.

We found 146 DEGs with the same numbers of up‐ and downregulated genes between the two groups. The list of DEGs and log_2_FC and the FDR‐adjusted *p* values of each gene were present in Supporting Information S1 (Table S4). Then, the DEGs were submitted for GO and KEGG analyses, and the top 10 GO terms in each BP (biological process), CC (cellular component), and MF (molecular function) were listed (Figure 7A). The results exhibited that these DEGs were mainly enriched in the BP, including epidermis development, skin development, epidermal cell differentiation, and keratinocyte differentiation. Similarly, cornified envelope, collagen‐containing extracellular matrix, and apical plasma membrane, which are mainly related to epidermal structure were most enriched in CC. As for MF, receptor‐ligand activity, signaling receptor activator activity, and enzyme inhibitor activity were primarily enriched. In the KEGG pathway annotation and enrichment analysis, we found that enriched pathways were associated with cytokine–cytokine receptor interaction, pancreatic secretion, and protein digestion and absorption (Figure 7B). Moreover, these targets were significantly enriched in many pathways related to cancer and immune function, not only were they involved in cytokine–cytokine receptor interaction, but also antifolate resistance, IL‐17 signaling pathway, NF‐kappa B signaling pathway, toll‐like receptor signaling pathway, and so on as well. The circle diagrams of GO and KEGG making the enrichment of up‐ and downregulated gene clusters much more intuitive were shown in Supporting Information S1 (Figure S9).

### 3.9 Drug screen and network of target genes construction

Sensitivity to common antitumor drugs (expressed in terms of IC50) was compared between two groups to determine potential treatment modalities for ESCA (Supporting Information S1: Figure S10). In addition, to screen potential compounds and drugs, the up‐ and downregulated DEGs of the two groups in ESCA were submitted for cMAP analysis. Alprostadil, pyrithyldione, tocainide, vancomycin, mefexamide, syrosingopine, metrizamide, perphenazine, and adenosine phosphate were screened out as the potential targets (Supporting Information S1: Table S5). Considering the cMAP scores, poisonous effects, and clinical applicability of these small molecule drugs synthetically, we chose PPZ for the further analysis (The number of evidence *n* = 5, low toxicity and side‐effect).

**Figure 8:**
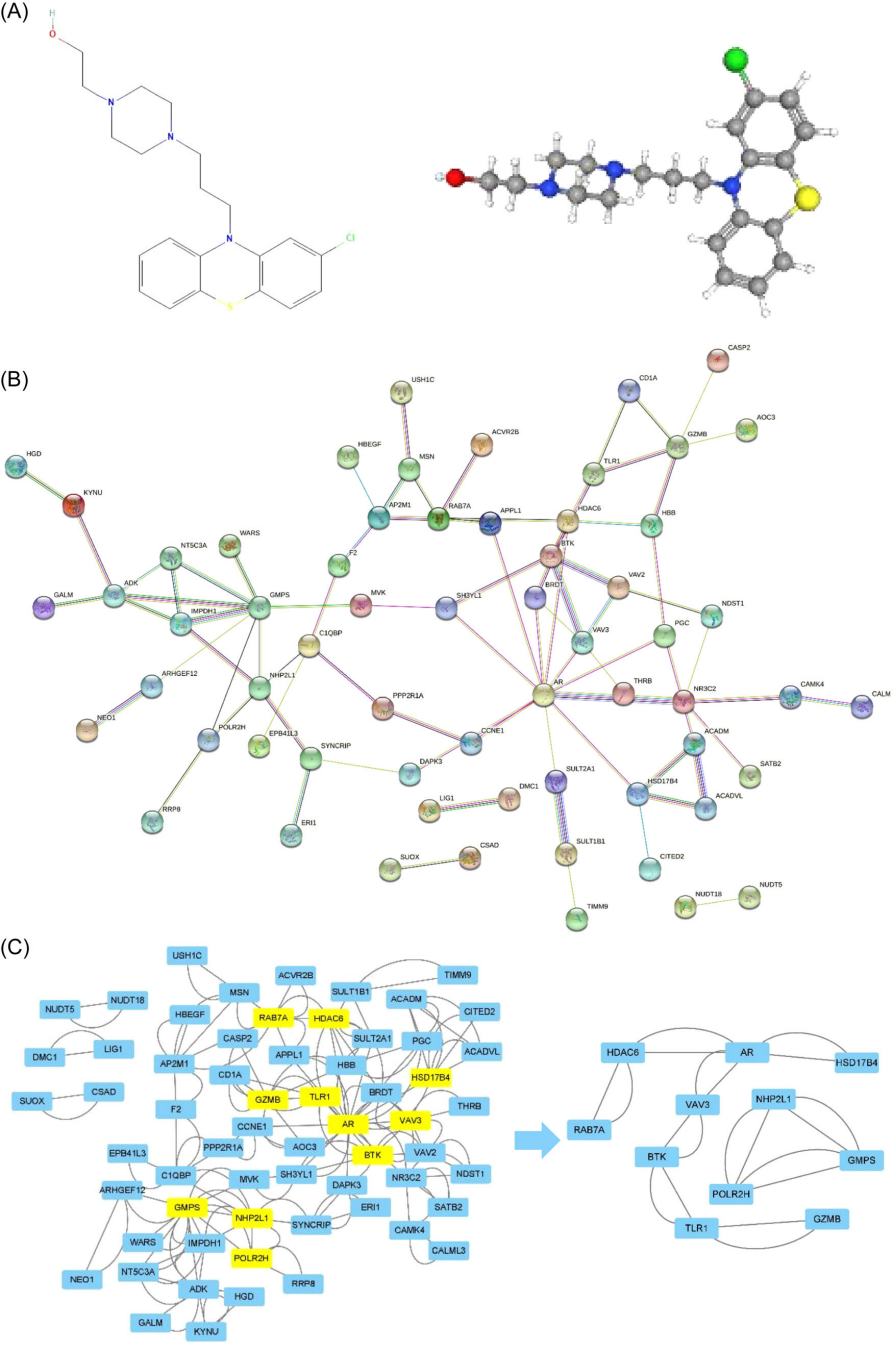
Structures and genetic networks of PPZ. (A) Two‐ and three‐dimensional chemical structure diagrams. (B) Protein–protein interaction (PPI) network. (C) Core targets of PPI network. PPZ, perphenazine.

With the help of PubChem, we obtained the two‐ and three‐dimensional chemical structures of PPZ (Figure 8A). To reveal the direct target of PPZ, the 3D_SDF structure was submitted to the PharmMapper, “Druggable Pharmacophore” Model was selected, and then the top 300 potential targets ranked by normalized fit scores in descending order were listed. Subsequently, we used multiple databases such as Uniprot, Ensembl, and so on to add gene symbols for potential targets, and finally obtained 77 target genes (Supporting Information S1: Table S6). Because the solitary proteins do not interact with other proteins, we hid them in the construction of a PPI network based on the targets of PPZ. Ultimately, a network containing 62 nodes and 156 edges was established (Figure 8B). In PPI network, a target node can be influenced by other nodes. The influence is represented by the edges, more edges in the current target node, results in the node occupying an increasingly significant position in the network. To find the key nodes, we analyzed the topological properties of the nodes in the PPI network via cytoscape_3.9.0. Accordingly, 11 key nodes (*HSD17B4, AR, HDAC6, RAB7A, VAV3, BTK, TLR1, GZMB, NHP2L1, POLR2H*, and *GMPS*) were identified (Figure 8C).

### 3.10 GO and KEGG enrichment analysis of PPI targets

**Figure 9:**
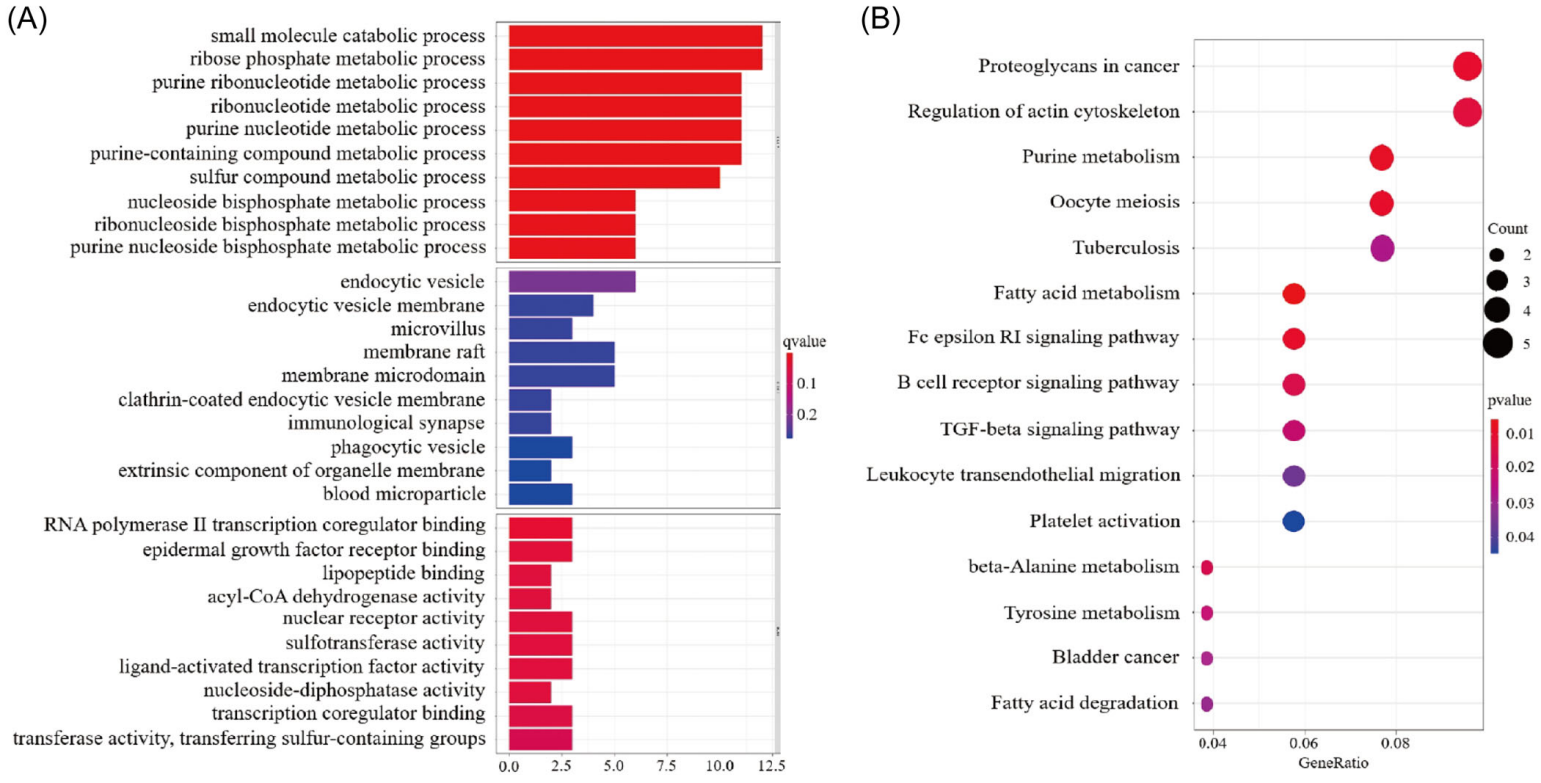
The enrichment analysis of drug targets. (A) GO functional enrichment analysis. (B) KEGG pathway enrichment analysis. Circle color indicates *P*. Size represents the gene number enriched in the pathway. GO, gene ontology; KEGG, Kyoto Encyclopedia of Genes and Genomes; TGF, transforming growth factor.

To further explore the differential functions and signaling pathways of the PPI targets, GO and KEGG enrichment analyses were conducted. Accordingly, the data showed that most of the drug targets were enriched in metabolism‐related processes (Figure 9A) and some pathways related to immunization process including Fc epsilon RI signaling pathway and B cell receptor signaling pathway were identified (Figure 9B). We also found that some tumor‐associated pathways including proteoglycans in cancer and transforming growth factor β (TGF‐β) signaling pathway.

According to the topological analysis of the PPI network and KEGG enrichment analysis, we found that *Vav3* (Vav guanine nucleotide exchange factor 3), *Btk* (Bruton tyrosine kinase), and *AR* (androgen receptor) were not only the key nodes in network but *Vav3* and *Btk* were also enriched in immune‐related pathways, such as Fc epsilon RI signaling pathway and B cell receptor signaling pathway. Meanwhile, *Vav3* is a signal molecule in proteoglycans in cancer that is considered an important tumor‐associated pathway and *AR* is enriched in oocyte meiosis, a pathway related to cell division. Thus, these three targets were chosen to confirm the accuracy of the above analysis.

### 3.11 Drug molecular docking

**Figure 10:**
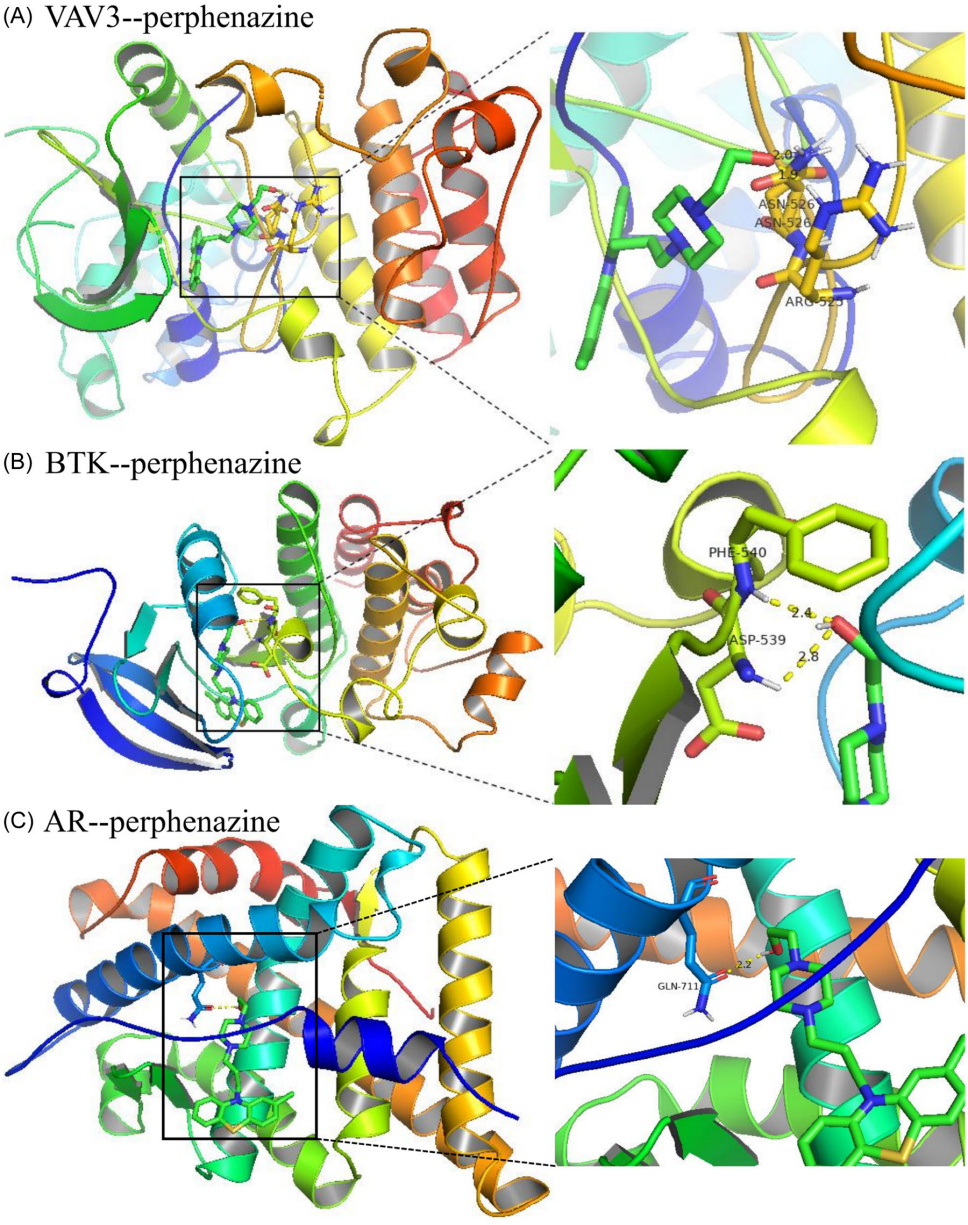
Docked complexes of PPZ with VAV3 (A, PDB ID: 2D86), BTK (B, PDB ID: 3GEN), and AR (C, PDB ID: 1E3G). The left views depict three‐dimensional docked pose of ligands with the target proteins and the right views are specific pics, showing the interaction for hydrogen bonds formed between PPZ and amino acids. AR, androgen receptor; Btk, Bruton tyrosine kinase; PPZ, perphenazine; Vav3, Vav guanine nucleotide exchange factor 3.

To determine the possible bindings of PPZ with the identified core targets, molecular docking analysis was performed (Figure 10). Molecular docking seeks to find the lowest energy conformation by combining the ligand and the receptor in their active area. With the affinity less than −5.0 kcal/mol applied as the screening standard, we found that the docking affinity of these targets with PPZ were all less than the standard (*AR* = −7.1, *Btk* = −8.2, and *Vav3* = −8.0 kcal/mol), indicating that the docking structures were relatively stable. Our results showed that the autodocking complex structures of PPZ and these core targets were similar; all of them were combined by forming hydrogen bonds between the hydroxyl group of PPZ and amino acid residues. The PPZ was stabilized by two hydrogen bonds with *Vav3* residues ARG‐525 and ASN‐526, same with *Btk* (The two amino acid residues were PHE‐539 and PHE‐540, respectively). As for *AR*, the PPZ was coordinated through one hydrogen bond with residues GLN‐711. These hydrogen bonds not only determined the positions of the compound in active area of three‐dimensional space but also further stabilized the docking.

### 3.12 Discussion

ESCA continues to pose a significant threat to healthcare systems around the world.^21^, ^22^ To diagnose the disease, the TNM staging system is still preferred as guidance in determining treatment plans as well as predicting prognosis. Nevertheless, the prognosis and clinical outlook of each patient still differ despite being of the same stage and having undergone similar treatment, due to both molecular and genetic cancer heterogeneity.^23^ Hence, it is crucial to identify molecular biomarkers of high specificity and sensitivity, to have early detection of ESCA, to monitor response to treatment, and to improve prognosis. The significant roles of the innate and the adaptive immune systems in the initiation and development of cancer have been affirmed by gathered evidence,^24^, ^25^ and immunotherapy currently shows clear clinical benefits in various cancers.^26^ Thus, considerable advancement of new and specific targeted therapy methods, particularly in combination therapy, is feasible with extended study concerning the link between IRGs' connection and tumorigenesis as well as tumor progression.^27^

In our study, the immunogenomic landscape of ESCA has been methodically assessed in regard to the TCGA database's gene expression profiles. No sole biomarker can be utilized in the detection of cancer, as it fails to achieve the required sensitivity and specificity.^28^ Therefore, we constructed successfully a 15‐IRGs prognostic signature and found that this signature was highly reliable for prognosis prediction and could offer incremental clinical value over traditional staging system, which can ameliorate treatment decisions. Moreover, the 15 crucial genes might act as prognostic factors of ESCA. For instance, previous studies have demonstrated a high expression of lncRNA *ELFN1‐AS1* in pancreatic cancer cell lines and tissues, as well as a notable increase in cancer cell apoptosis and growth arrest via the gene knockdown of *ELFN1‐AS1*.^29^ In glioblastoma cells, upregulation of both the *APLN* and the Apelin receptor (*APLNR*) allow control over the invasiveness of tumor cells take place.^30^ Cancer cells fail to survive through the ubiquitination and destabilization of *YAPI* by certain protective genes, such as *STUB1* and *CTF1*, specifically the E3 ubiquitin ligase *STUB1*.^31^ Cardiotrophin‐1 (*CTF1*) is a type of interleukin‐6 that has well‐established protective and hypertrophic actions towards cardiac myocytes,^32^ but the connection between *CTF1* and cancer is still not well‐explored. Given how *CTF1* functions, it is hypothesized to reduce the cardiotoxicity of tumor sufferers after treatment and protect other organs such as the liver, kidney, or nervous system.^33^, ^34^, ^35^ Thus, the prognostic signature founded upon these 15 genes is well‐established and reliable.

In gene mutation analysis, similar mutation frequencies were displayed in the two patient groups, and mostly missense mutation was the type of mutation found in patients with ESCA. Exploring the causes of differences in prognosis may require attention to the mutation locations and types of high‐frequency mutated genes.^36^, ^37^ *TP53* has the highest mutation rate, and the resistance of *p53* protein to proteolytic degradation by E3 ubiquitin ligases is frequently caused by missense mutations, guaranteeing a large amount of stable mutant *p53* protein.^38^ On the other hand, around 42% of tumors undergo mutations in *TP53*, displaying a less desirable prognosis for certain cancers.^39^ That said, numerous variables including the type of cancer, clinical stage, study size, and mutation status of *TP53* can also influence prognostic determinations. It is not guaranteed that *p53* signaling status in a cancer cell can always be indicated by *TP53* mutation status.^40^, ^41^ Hence, a more precise prediction in prognosis may be obtained from a flexible reference to the *TP53* gene's mutation in the readout of *p53*'s role in human cancers.

A large number of functions related to metabolization and proliferation were enriched among patients in the high‐risk group, possibly because immune dysregulation in the tumor microenvironment and highly proliferative human ESCA cells rely on metabolic processes to enhance the uptake of lipids and other substances to maintain cell hyperplasia.^42^ But among patients with low risk, these DEGs were enriched in processes related to cornification and immunization. Antiapoptotic and antinecroptotic pathways can be activated by keratinocytes for the prevention of premature cell death in the stage of terminal differentiation,^43^ whereas cellular immune responses are the key to effective cancer immunosurveillance. Notwithstanding, keratinocytes might cause the appearance of malignant proliferation, tumor infiltration, and other phenomena in the low‐risk group, but there were still some immunoreactions to restrain oncology progress, such as T cells being the most crucial cancer cell killing tool in the immune system.^44^ One hundred forty‐six DEGs identified from high‐ and low‐risk patients were predominantly involved in epidermis development, skin development, and keratinocyte differentiation in biological process enrichment analysis, cornified envelope was most enriched in the cellular component. In the enrichment analysis of the KEGG pathways, it was discovered that three pathways displayed significant statistics, such as cytokine–cytokine receptor interaction, pancreatic secretion, and protein digestion and absorption. GO enrichment analysis confirmed the results of GSEA, which showed that the cornification and apoptosis of keratinocytes were disordered and delayed, respectively.

By the online analysis websites cMAP and PharmMapper, a small molecule drug named PPZ was screened. PPZ could inhibit the expression of high‐risk genes in our model and we momentarily got its molecular targets. Currently, there are extensive studies and applications of increasing numbers of drugs besides immune checkpoint inhibitors, including mTOR (mammalian target of rapamycin) inhibitors, which may potentially treat malignant tumors.^45^ The same can be said of PPZ which has always been a psychotropic drug. Recent research suggests that PPZ and prochlorperazine not only possess sedative, antiemetic, and anticancer activity but also alter multidrug resistance in oncotherapy.^46^ In cutaneous melanoma, the sensitivity of cancer cells towards treatment can be restored by PPZ.^47^ In endometrial cancer, PPZ displayed brilliant cell proliferation and migration inhibition in Ishikawa (ISK) and KLE cell lines.^48^ The results of the GO and KEGG enrichment analysis of drug targets propose that PPZ exerted effects in metabolic processes, anti‐inflammatory activities, immunoregulation, and related signaling regulation pathways such as *BCR* (B cell receptor), *FcεRI* (Fc epsilon RI), and proteoglycans in cancer‐directed anti‐ESCA. Furthermore, core molecules or genes, such as *HSD17B4, AR, HDAC6, RAB7A, VAV3, BTK, TLR1, GZMB, NHP2L1, POLR2H*, and *GMPS*, could regulate the anti‐ESCA action of PPZ. Through an analysis of molecular docking, good binding activities of PPZ with *VAV3, BTK*, and *AR* were found, thus suggesting the ability of PPZ to cause *VAV3* and *BTK* to regulate immune responses and to restrain cell proliferation by *AR* in ESCA. Hence, the small molecule drugs stipulated in this study have the potential to supply new ideas and to act as a basis for future studies on clinical drugs. Our belief is placed on the ability of adjuvant supplementation of PPZ to improve the therapeutic efficacy of present clinical antineoplastic agents and immunotherapy for ESCA treatment.

## 4 CONCLUSION

In summary, we succeed in structuring the immune‐related prognostic model for ESCA based on TCGA, which could be used to distinguish and predict patients' survival outcome, and screening a small molecule drug named PPZ by cMAP. Then, the network pharmacology and computational findings highlight the metabolic process and immunoregulation as crucial targets/pathways of PPZ adjuvant treatment in ESCA, and the potent pharmacological targets against ESCA were determined. Finally, molecular docking was performed to explore and verify the drug‐core targets interactions, allowing for extensive clinical trials in the future. However, there are also certain limitations, prospective studies are needed to further validate its analytical accuracy for estimating prognoses and to test its clinical utility in individualized management of esophageal cancer.

## AUTHOR CONTRIBUTIONS

Baosheng Zhao and Yuzhen Liu designed the study. Pengju Qi performed bioinformatics research. Pengju Qi, Bo Qi, Chengwei Gu, and Shuhua Huo sorted data. Pengju Qi and Yuzhen Liu wrote the paper. Pengju Qi, Xinchen Dang, Yuzhen Liu, and Baosheng Zhao revised the manuscript. All authors read and approved the submitted version.

## CONFLICT OF INTEREST STATEMENT

The authors declare no conflict of interest.

## ETHICS STATEMENT

The present study was conducted in accordance with the declaration of Helsinki, and approved by the Institutional Review Board for Human Research of the First Affiliated Hospital of Xinxiang Medical University. Informed consent was obtained from every patient participating in this study. Institutional Review Board approval is not needed for this study since it does not involve any animals.

## Supporting information

Supporting information.

## Data Availability

The data sets presented in this study can be found in online repositories. The names of the repositories and accession numbers can be found in the article. Further inquiries can be directed to the corresponding authors.
