# Supplementary material for: Construction of an immune‐related prognostic model and potential drugs screening for esophageal cancer based on bioinformatics analyses and network pharmacology
Source: Immun Inflamm Dis. 2024 May 28;12(5):e1266. doi: 10.1002/iid3.1266 (PMC11131936; doi:10.1002/iid3.1266)
Supplement: Supplementary file 1 — Supporting information. [file IID3-12-e1266-s001.pdf]

Supplementary materials and methods

2.1 Patients and esophageal cancer tissue collection

In this study, thirty patients with esophageal squamous cell carcinoma (ESCC) diagnosed by histopathological examination between 2014 and 2016 were enrolled in the Department of Thoracic Surgery, the First Affiliated Hospital of Xinxiang Medical University. Notably, these patients had not received any radiotherapy or chemotherapy prior to the surgical procedure performed. All ESCC and adjacent tissues were obtained from surgical resection and immediately stored in -80°C. Total RNA was extracted from tumors using Trizol reagent (Ambion, Carlsbad, USA).

2.2 Extraction of IRGs

2,660 IRGs were downloaded from IMMPORT (https://www.immport.org/) and InnateDB (https://www.innatedb.ca/), including chemokines and their receptors, cytokines and their receptors, interferons, interleukins, etc. By means of the R package "limma" to analyze and extract the acquired mRNA matrix, the expression levels of IRGs in the patients were obtained.

2.3 Data acquisition

The results of simple nucleotide variation, transcriptome profiling of RNA expression (FPKM) and clinical data form 160 ESCA patients and 11 normal controls were gathered form TCGA (https://portal.gdc.cancer.gov/), a valuable data source for analyzing complex cancer genomics and clinical parameters. In addition, the genome annotation file of human (Homo_sapiens.GRCh38.104.chr.gtf) was obtained from Ensembl (https://asia.ensembl.org/index.html). The above data are publicly available.

2.4 WGCNA

The R package “WGCNA” in R-language provides a series of functions to create a weighted gene co-expression network, which can evaluate the connection strength between genes more flexibly. In order to make the network conform to the scale-free characteristic, it is necessary to select an appropriate weight value. By first clearing missing and outlying values, then using a function named “pickSoftThreshold” in the “WGCNA” package to calculate the weight value, the soft threshold (power) of β=3 was finally selected to establish the co-expression network. The calculation formula of adjacency matrix is $A_{mn}=((1+S_{mn})/2)^{\beta}$. In order to exclude the error induced by background noise and pseudo-correlation, the adjacency matrix was converted into topological overlap matrix (TOM) to describe the association strength and to calculate dissimilarity (dissTOM=1-TOM) between the genes. TOM was applied as an input for the hierarchical clustering analysis of genes, and the algorithm “DynamicTreeCut” was applied to identify network modules, minModuleSize=40, and cutHeight=0.3. Finally, the correlation test was used out with clinical data and the module-trait relationships were visualized by using the directive “labeleheatmap”.

2.5 Construction and evaluation of the ESCA prognostic signature

In order to identify possible prognostic IRGs, further investigation of the mRNAs selected for constructing the immune-related prognostic signature was conducted via application of univariate Cox regression analysis. According to the co-expression relationship of mRNA and lncRNA, the corresponding lncRNAs were selected via using the R package “limma”. The possible prognostic lncRNAs were screened in the

same way. Before constructing the prognostic model, we used an instruction "createDataPartition" in the R package "caret" to randomly divide the patients into training set and validation set. LASSO can select variables and estimate parameter simultaneously, thus it can better solve the multicollinearity problem in regression analysis and is used as a biased estimation tool for data with complex collinearity [24]. LASSO Cox regression analysis was performed in this study to reduce the gene number and to select the risky signatures of mRNA and lncRNA in order to create the prognostic model. Finally, the risk scores of every patient from TCGA were calculated based on these signatures, and all patients were separated into high- and low-risk groups with the median score as cut-off value. Risk score was calculated as follows: $Risk\ score = \sum_{i=1}^{n} Coefi * expi$, where 'coef' was the risk coefficient and 'exp' was the expression level.

Using R package 'ggplot2' and 'scatterplot3d' to draw principal component analysis (PCA) respectively, which could assess the efficiency of cohort clustering. Survival analysis for each group was evaluated by the Kaplan–Meier curve and log-rank test. The receiver operating characteristic (ROC) curve and the area under the curve (AUC) were drawn using the R package "timeROC". An AUC>0.75 was recognized as an excellent predictive value. Then, in R-language (4.1.0), we integrated the risk score with existing clinical and pathologic characteristics for univariate and multivariate Cox regression and heatmap analysis by the R package "survival", "ComplexHeatmap" and "RColorBrewer".

2.6 Differential analysis between high- and low-risk groups

The ESTIMATE algorithm was used to compute the immune, stromal, and ESTIMATE score of each ESCA patient, and correlations between IRS and immune cell infiltration (ICI) were analyzed by multiple software such as XECLL and CIBERSORT. Gene set enrichment analysis (GSEA) was carried out for differentially expressed genes (DEGs) in high- and low-risk groups by using the GSEA_4.1.0 software, c5.go.v7.4.symbols.gmt (Gene ontology) as gene sets database, and the number of stochastic combinations was set to 1000. The up- and down-regulated gene analyses of Gene Ontology (GO) and Kyoto Encyclopedia of Genes and Genomes (KEGG) pathway were analyzed using the R package "clusterProfiler", with $P < 0.05$ indicating statistical significance. In terms of gene mutation, downloading the somatic mutation data of varscan software and calculated tumor mutational burden (TMB) by Perl script. The formula is: TMB = (total number of somatic mutation) / (length of coding region), unit is mutations/mb. The R package "maftools" was used to visualize the mutation data of high- and low-risk groups. Finally, to explore potential clinical drugs for treating ESCA, we used R package 'pRRophetic' to predict the half inhibitory concentration (IC50) values of anticancer medications.

2.7 Drug screen

Potential small molecule drugs applied for treatment of ESCA were forecasted by using the connectivity map (cMAP, https://portals.broadinstitute.org/cmap/) including of genome-wide transcriptional expression data from small molecule drugs. The DEGs' symbols were converted into corresponding probe IDs and input into cMAP database for searching drugs that could counter the change of gene expression. The

negative score indicates that small molecule compounds or drugs have an antagonistic relationship with the biological processes or states of differential genes, suggesting that these genes and drugs have opposite mechanisms of action, and may be potential therapeutic drugs for ESCA. Among drugs with antagonistic effects, PPZ had more evidence (n = 5) and fewer toxic side effects, so we chose this drug for the further analysis.

2.8 Network pharmacology analysis

The 3D_sdf file of small molecule was acquired from the PubChem database (https://pubchem.ncbi.nlm.nih.gov/), and then input to the online program PharmMapper (http://www.lilab-ecust.cn/pharmmapper/) to simulate the ligand-target docking. The range was set to "Druggable Pharmacophore Models (v2017, 16159)", while the rest of parameters remained unchanged. After the task was completed, all molecular targets were re-annotated (add gene symbols) by multiple databases such as Uniprot, PDB, and Ensembl and et al, empty matches were eliminated. We imported the annotated and summarized target list into the STRING website (https://cn.string-db.org/), selected the "multiple proteins" project, and defined the species category as "Homo sapiens". After generating the protein-protein interaction network, we saved the result of image and downloaded the TSV file, then input these data to cytoscape_3.9.0 for optimization and core targets extraction. The protein structure was obtained from the PDB database (https://www.rcsb.org/) and removed ligands and non-protein molecules by the pymol software. MM2 force field optimization was conducted using the three-dimensional structure of the compound

downloaded by the Chem3D. Raccoon software was used to create the Protein Data Bank, Partial Charge, &Atom Type (PDBQT) structure files necessary for virtual screening. MGLTools 1.5.7 of Autodock Vina software as the supporting tool was used to process the corresponding protein, the hydrogenation, the Gasteiger charge for merging nonpolar hydrogen atoms, and so on. The original PDB file format was converted to the PDBQT file format recognized by the Autodock Vina program, providing a ligand basis for molecular docking (Forli S et al., 2016). Finally, after setting the Grid Box coordinates, docking and visualization were done by the software of vina and pymol respectively (Trott O et al., 2016).

2.9 Statistical Analysis

All statistical analyses were performed on R software (4.0.1). The Chi-square test was used to compare the clinicopathological features between different groups. Log-rank test was conducted to analyze the significance of the Kaplan-Meier survival differences between groups. Univariate and multivariate Cox regression analyses were used to identify the independent prognostic factors for patients with ESCA. The Wilcoxon test was performed to assess immune cell infiltration levels between risk groups. Expression levels were analyzed using the $2-\Delta\Delta CT$ method, each assay was repeated three time, and statistically significant difference was set at $P < 0.05$. Data are presented as with representing a statistically significant result.

Supplementary Results

Construction of the ESCA prognostic signature

Risk Score = 0.3066*Express quantity of ELFN1-AS1 + 0.1046*Express quantity of JPX + 0.5041*Express quantity of AC127024.3 + 0.0272*Express quantity of CRABP1 + 1.0331*Express quantity of FABP9 + 0.2874*Express quantity of HSPD1 + 0.1944*Express quantity of PSMC6 + 0.1423*Express quantity of PSMD10 + 0.0755*Express quantity of APLN + -0.1463 Express quantity of CTF1 + 0.4807*Express quantity of STC2 + -0.2464*Express quantity of STUB1 + 0.0478*Express quantity of PMAIP1 + -0.445*Express quantity of CDK9 + 0.28*Express quantity of SUGT1.

Independent prognosis analysis

We conducted an independent univariate and multivariate Cox regression analysis with the fifteen genes. Because of the high correlation between stage and pathological stage-TNM in the clinical feature, we finally selected stage instead of TNM for the Cox regression analysis. For TCGA training set and validation set data, univariate and multivariate Cox analyses showed that the stage and IRS revealed significant prognostic effect in univariate analysis. In multivariate analysis, only IRS could be a significant independent prognostic factor (**Supplementary Figure S4**). Furthermore, we included the information about smoking or drinking in the overall set, and found that the conclusion that the IRS is a significant independent prognostic factor was not affected (**Supplementary Figure S2D**). Even in the training set and validation set, tobacco and alcohol could not be used as prognostic indicators ($P > 0.05$) and had

no effect on IRS (In univariate analysis, the *P* values of training and verification set were < 0.001 and 0.007 respectively, and the *P* values were 0.003 and 0.024 in multivariate analysis respectively). Hence, the prognostic model based on 15 IRGs could be identified as an important indicator, and indeed be significant for clinical application in ESCA.

Model Comparison

In order to prove the value of immune-related genes in prognostic diagnosis, and to illustrate the advantages of multi-index prognostic model, we obtained the corresponding prognostic model from four published literatures (Guo et al., 2022; Jiang et al., 2023; Ren et al., 2023; Xiong et al., 2022;), in which both Guo and Xiong signatures were composed of immune-related mRNA. Through comparison with these four models, we found that the C-index and AUC of our risk model were higher, and the different prognoses of survival curves were also more significant (**Figure S5**), indicating our model has more prognostic value.

Supplementary Figures

Figure Captions

FigureS1. Weighted correlation network analysis for IRGs. Heatmap of the correlation between module genes and clinical phenotypes. The color of red and blue represents positive and negative correlation, respectively. The numbers at the top indicate correlation coefficients, and the numbers in parentheses represent *P*.

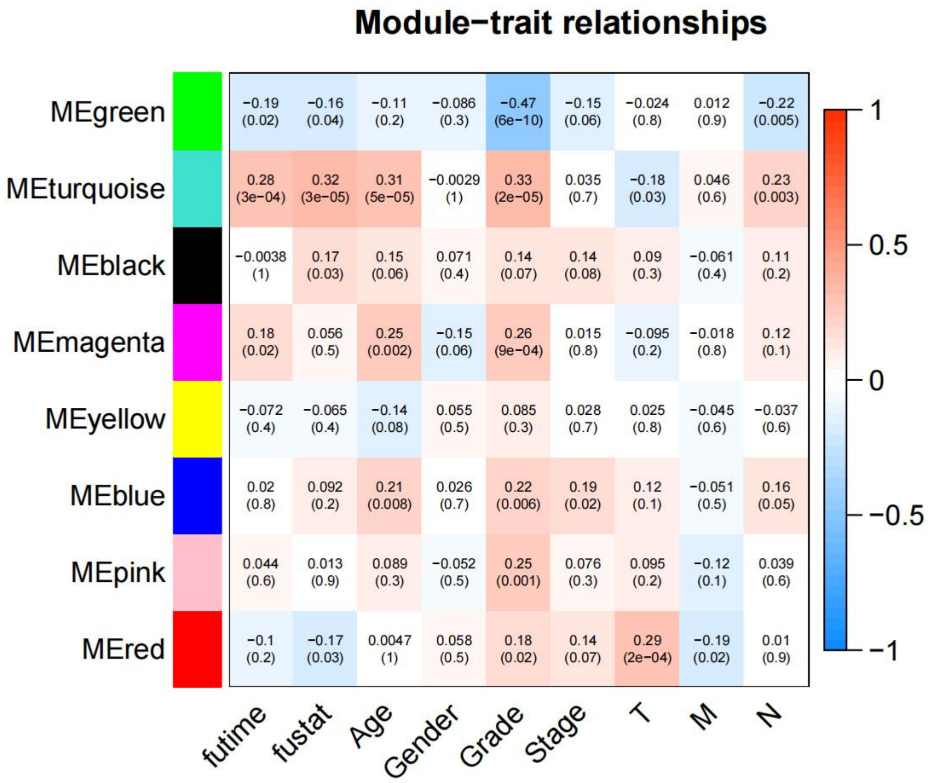

FigureS2. Topological structure analysis of soft threshold parameters **(A)** and Hierarchical clustering tree **(B)**.

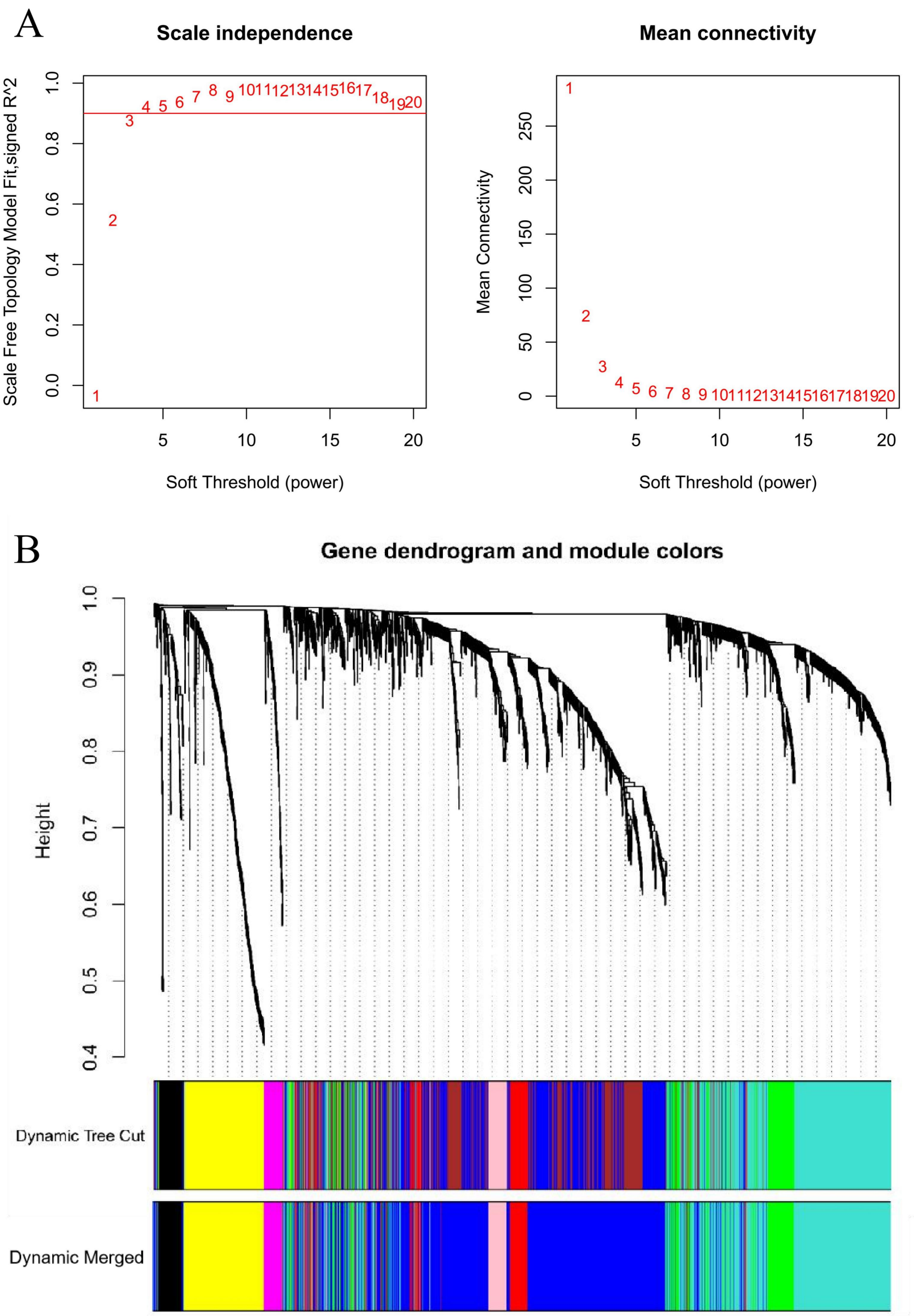

Figure S3. The quality validation of the prognostic model for all ESCA patients.

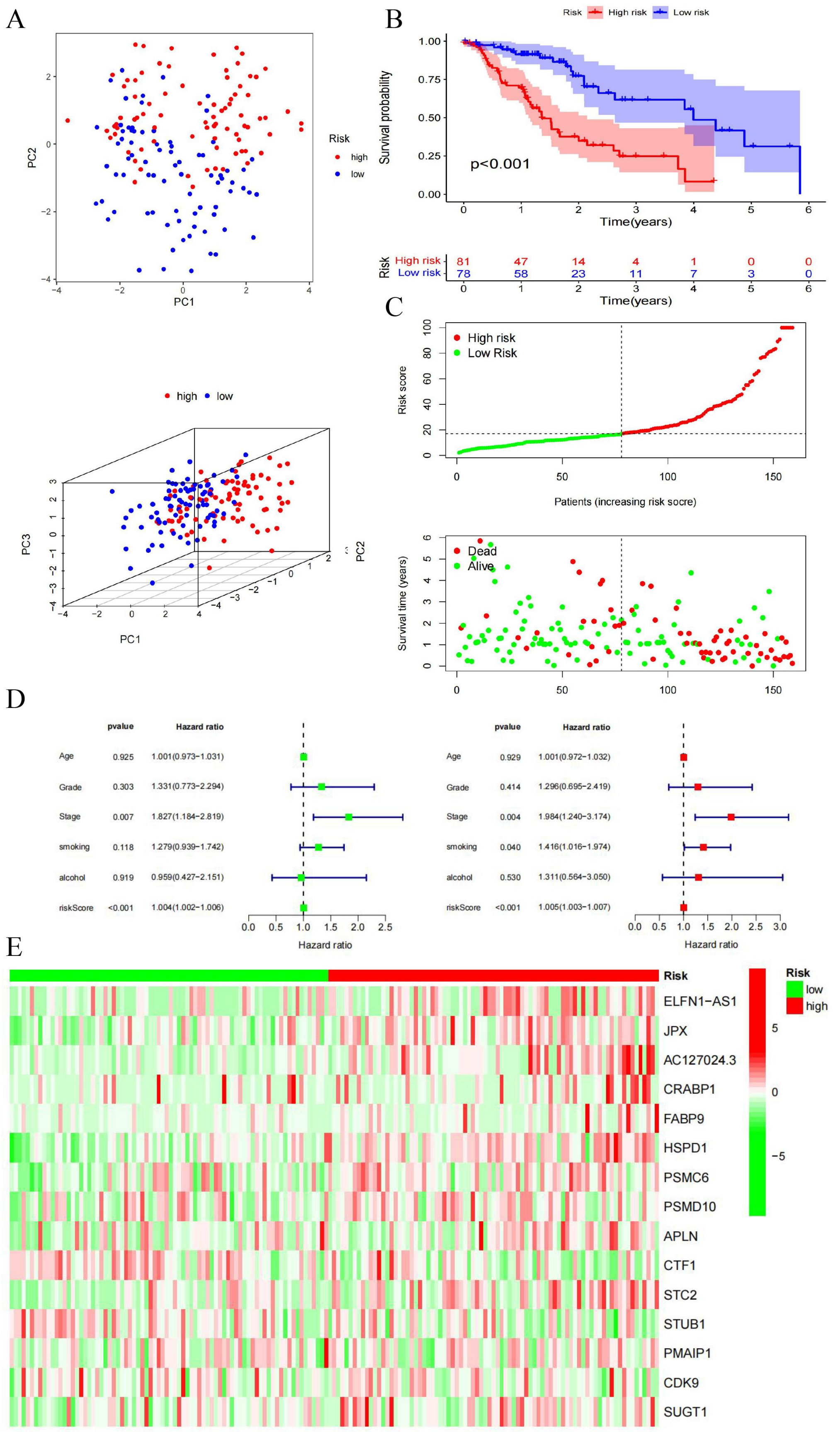

Figure S4. Forest plots show clinicopathological features that significant related to survival by univariate and multivariate Cox regression analysis. $P < 0.05$ is indicated significant.

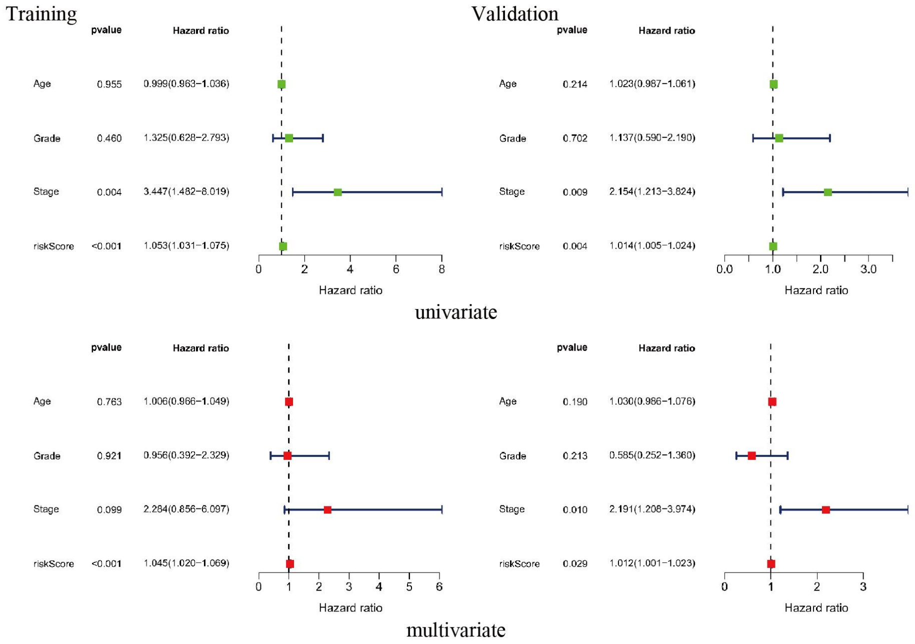

Figure S5. Model Comparison. The C-index of different prognostic models **(A)**. Time-dependent ROC curve and Kaplan-Meier survival curve of the fifteen-IRG signature **(B)**. Time-dependent ROC curve and Kaplan-Meier survival curve of the other different prognostic models **(C)**.

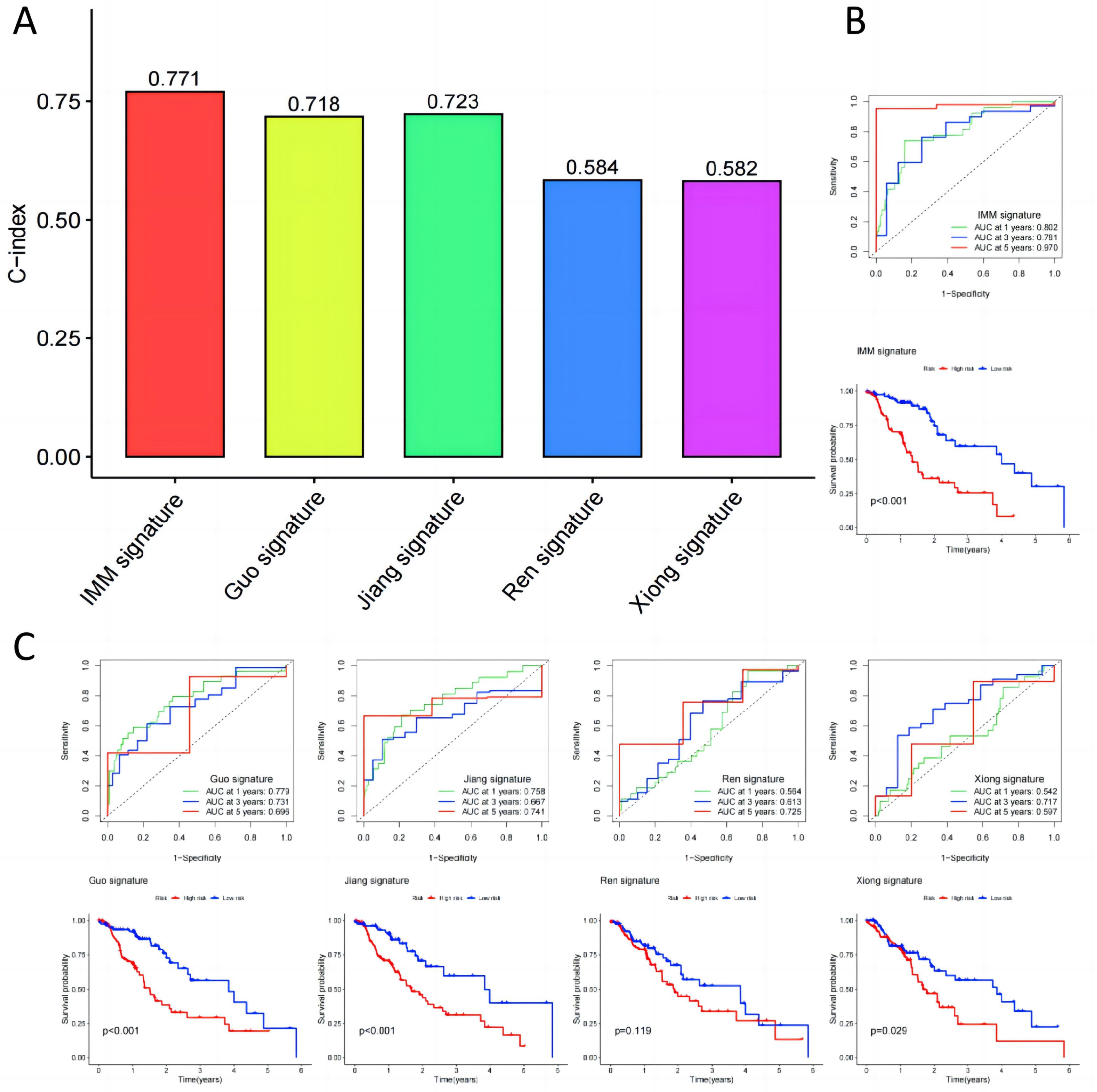

Figure S6. Differences in TME between the high- and low-risk groups. Comparison of the stromal score, immune score, and ESTIMATE score **(A)**. The correlation between risk score and immune cell infiltration in ESCA patients **(B)**.

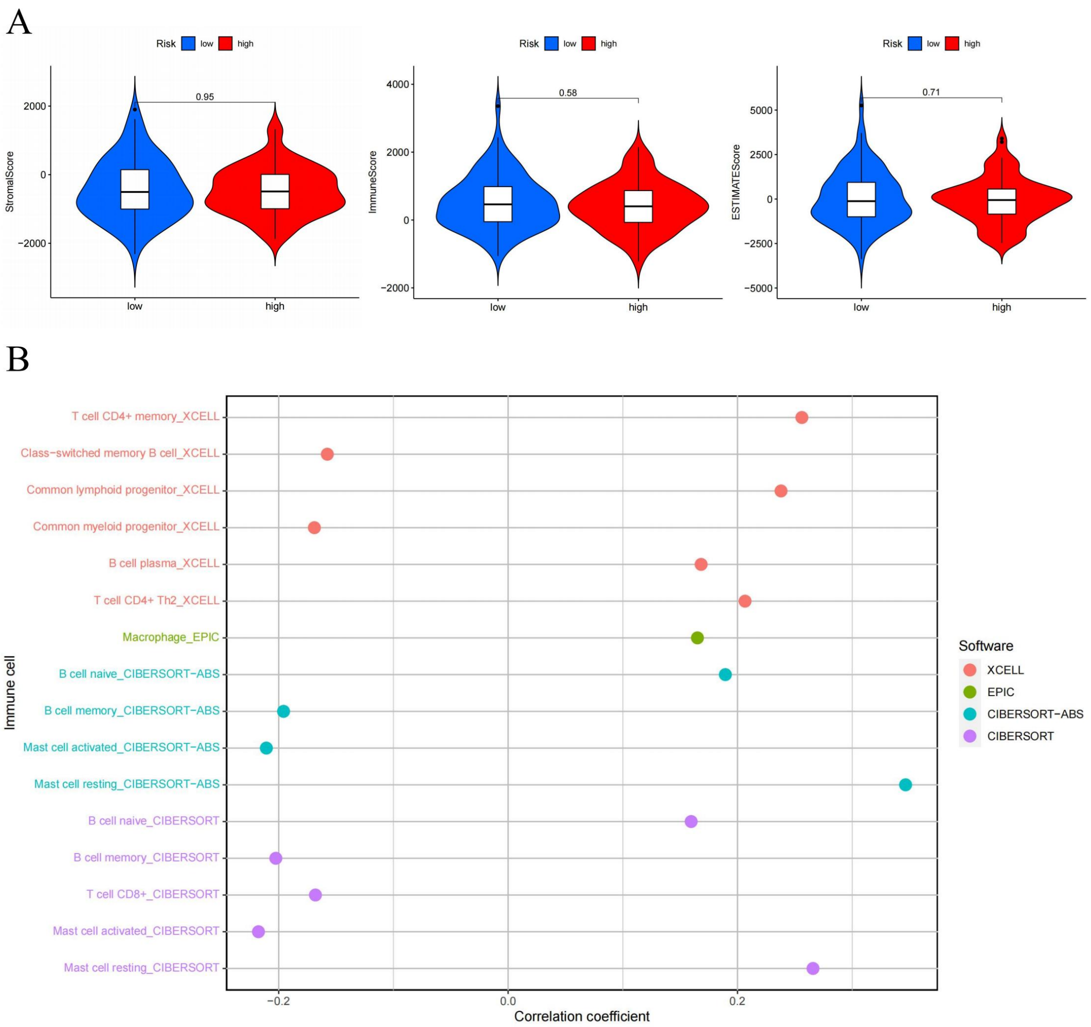

FIGURE S7. The distribution of frequently mutated genes in the high- **(A)** and low-risk groups **(B)**. Annotations with different colors at the bottom refer to the various mutation types and the top bar chart present tumor mutational burden (TMB) per patient. The right bar chart shows the frequency of each gene mutation.

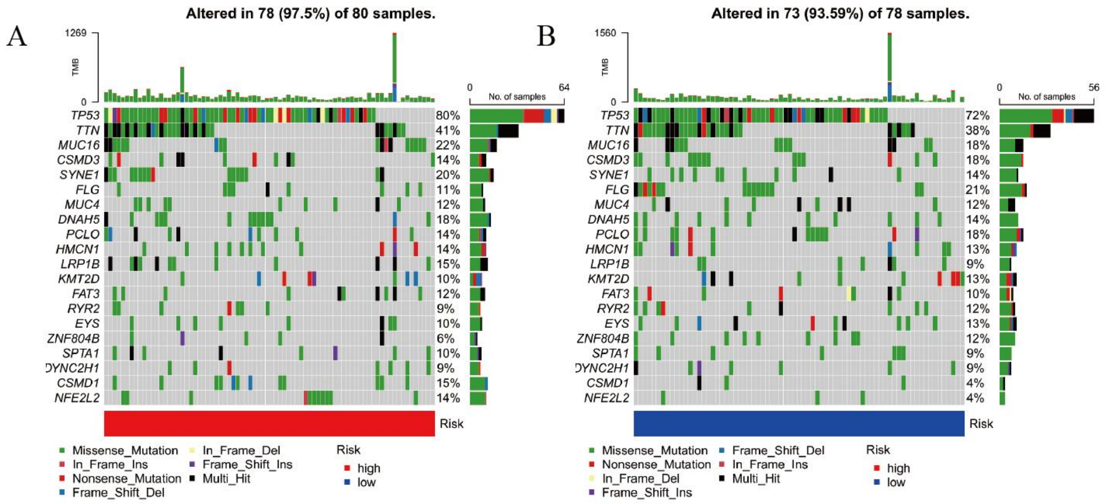

Figure S8. Analysis of tumor mutational burden.

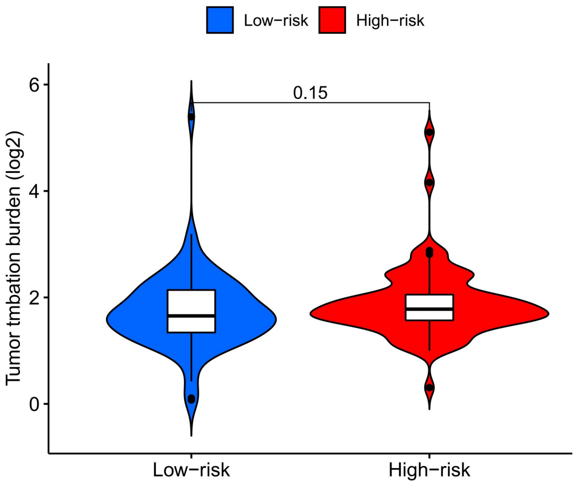

Figure S9. Enrichment analysis.

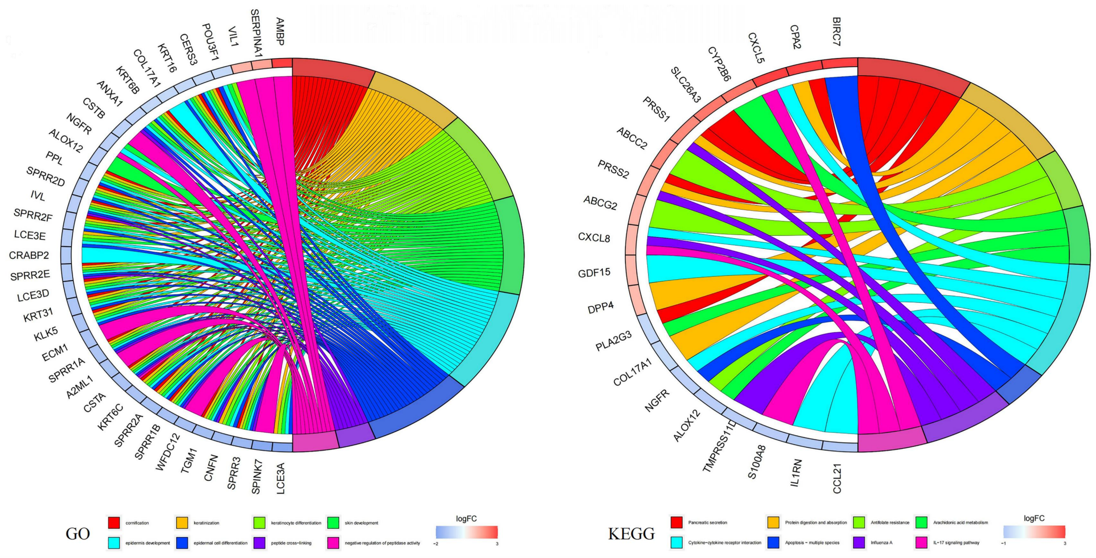

Figure S10. Estimated drug sensitivity in the high- and low-risk groups.

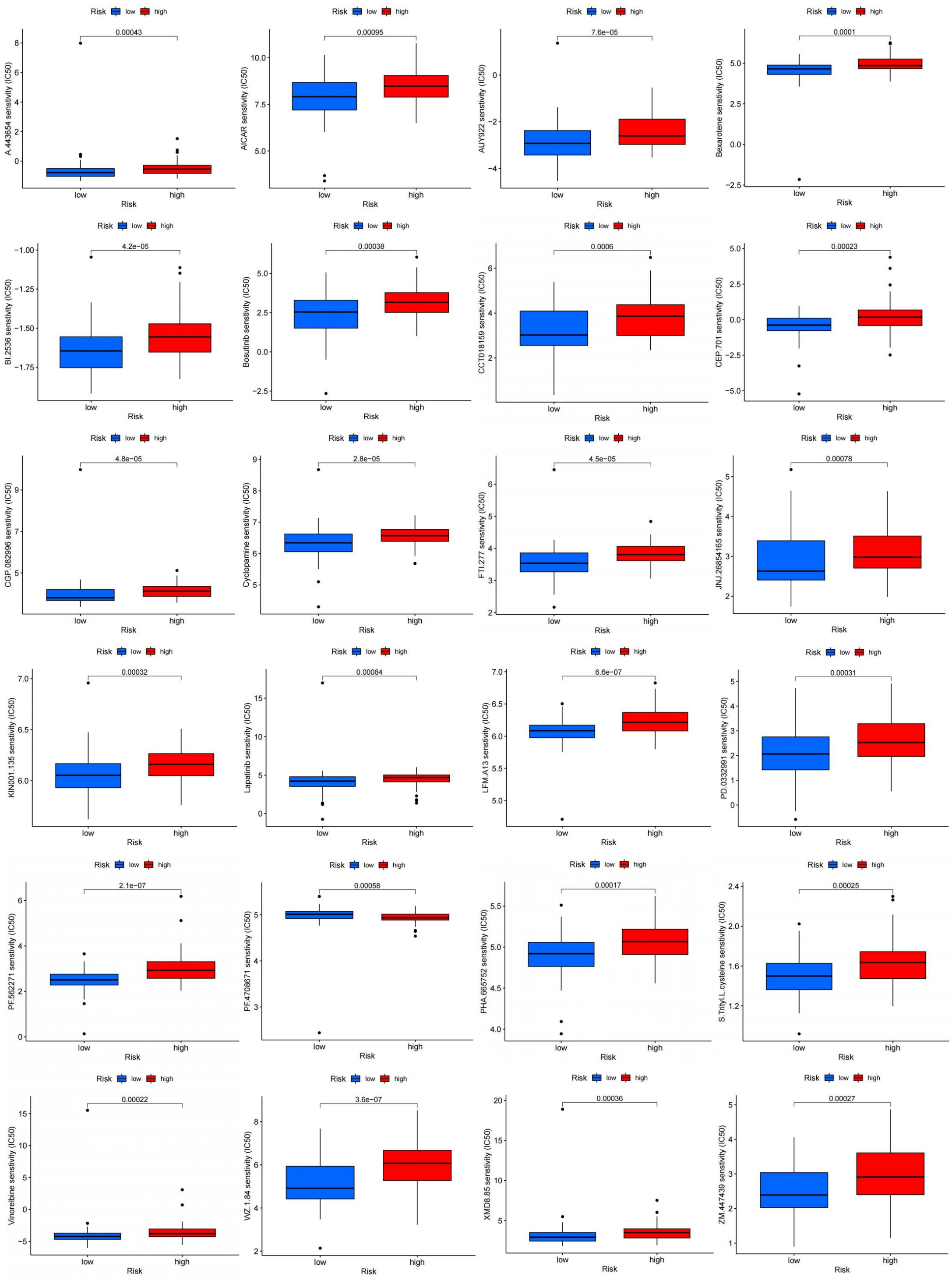

Supplementary Tables

Table S1. Clinical information of the 30 ESCC patients.

[illegible]	Surgery Date	Gender	Age (year)	Stage	T	N	Tumor size(cm)	Tumor location	Tumor type	Differentiation degree	Overall survival(mo
[illegible]	2014.01.10	Male	50	IIIB	T3	N1	5.1	upper	ulcerative	middle	14
[illegible]	2014.03.27	Female	67	IIIA	T2	N1	8.3	lower	ulcerative	middle	6
[illegible]	2014.05.05	Male	64	IIB	T3	N0	6.2	middle	medullary	middle	9
[illegible]	2014.05.06	Male	59	IIA	T2	N0	10	lower	medullary	middle	12
[illegible]	2014.05.31	Male	63	IB	T2	N0	5.6	middle	ulcerative	high	11
[illegible]	2014.07.15	Female	71	IIB	T3	N0	6.3	middle	medullary	middle-high	7
[illegible]	2014.09.04	Female	72	IIA	T2	N0	5.6	middle	ulcerative	middle	6
[illegible]	2014.09.15	Male	67	IIIB	T3	N1	8.4	middle	ulcerative	high	11
[illegible]	2014.09.25	Male	76	IIIB	T3	N1	4.8	middle	protruded	middle	12
[illegible]	2014.09.26	Male	69	IIIB	T3	N1	5	middle	constritive	low	13
[illegible]	2014.11.19	Male	66	IIB	T3	N0	4.7	upper	ulcerative	middle	13
[illegible]	2015.04.23	Male	62	IB	T2	N0	7	middle	ulcerative	high	12
[illegible]	2016.04.25	Female	70	IB	T1b	N0	2.7	middle	medullary	middle-low	6

[illegible]	Surgery Date	Gender	Age (year)	Stage	T	N	Tumor size(cm)	Tumor location	Tumor type	Differentiation degree	Overall survival(ye
[illegible]	2014.2.24	Male	65	IB	T1b	N0	4.3	middle	medullary	high	>8
[illegible]	2014.3.24	Male	64	IB	T1b	N0	2.6	middle	ulcerative	high	5
[illegible]	2014.4.15	Male	61	IIA	T3	N0	5.9	middle	ulcerative	high	7 (87 mout
[illegible]	2014.6.4	Female	59	IB	T1a	N0	3.9	middle	mushroom	middle-low	>7
[illegible]	2014.6.9	Male	70	IB	T2	N0	4.5	lower	medullary	high	7 (95 mout
[illegible]	2014.6.14	Male	57	IIB	T3	N0	2	middle	medullary	middle	>7
[illegible]	2014.6.28	Male	62	IIB	T3	N0	5	upper	medullary	middle	>7
[illegible]	2014.7.21	Male	64	IB	T2	N0	3.8	lower	ulcerative	high	>7

[illegible]	2014.7.22	Male	72	IIB	T3	N0	12.8	middle	medullary	low	>7
[illegible]	2014.8.4	Male	59	IIIA	T2	N1	3.5	lower	ulcerative	high	>7
[illegible]	2014.8.21	Female	65	IIA	T2	N0	5.3	middle	medullary	middle	>7
[illegible]	2014.9.20	Female	65	IC	T2	N0	5	middle	ulcerative	high	>7
[illegible]	2014.10.18	Male	68	IB	T1b	N0	5.6	middle	protruded	low	>7
[illegible]	2014.11.17	Male	61	IIIA	T2	N1	5.6	middle	medullary	middle	>7
[illegible]	2015.2.12	Female	58	IIIB	T3	N1	3	middle	medullary	middle-high	>7
[illegible]	2015.6.15	Male	66	IIIB	T3	N1	4.7	middle	medullary	middle	>6
[illegible]	2016.1.28	Female	48	IIIB	T3	N2	6.3	middle	ulcerative	high	>6

Table S2. Summary of clinical phenotypes in all patients

**Covariates**	**Risk**	**Total**	**high**	**low**	**chi**	***P***
Age	<=65	98(61.64%)	50(61.73%)	48(61.54%)	0	1
	>65	61(38.36%)	31(38.27%)	30(38.46%)		
Gender	FEMALE	23(14.47%)	11(13.58%)	12(15.38%)	0.0096	0.922
	MALE	136(85.53%)	70(86.42%)	66(84.62%)		
Grade	G1-2	81(50.94%)	31(38.27%)	50(64.1%)	8.4379	0.0037
	G3	43(27.04%)	29(35.8%)	14(17.95%)		
Stage	Stage I-II	84(52.83%)	32(39.51%)	52(66.67%)	12.1465	5.00E-04
	Stage III-IV	56(35.22%)	39(48.15%)	17(21.79%)		
T	T1-2	64(40.25%)	26(32.1%)	38(48.72%)	3.7066	0.0542
	T3-4	79(49.69%)	46(56.79%)	33(42.31%)		
M	M0	119(74.84%)	58(71.6%)	61(78.21%)	3.0895	0.0788
	M1	8(5.03%)	7(8.64%)	1(1.28%)		
N	N0	65(40.88%)	21(25.93%)	44(56.41%)	14.9015	1.00E-04
	N1-3	77(48.43%)	51(62.96%)	26(33.33%)		
smoking	NO	46(28.93%)	21(25.93%)	25(32.05%)	0.0359	0.8496
	YES	96(60.38%)	47(58.02%)	49(62.82%)		
alcohol	NO	46(28.93%)	28(34.57%)	18(23.08%)	1.8868	0.1696
	YES	110(69.18%)	52(64.2%)	58(74.36%)		

Table S3. Summary of clinical phenotypes in the training set and validation set

**Covariates**	**Training set**				**Validation set**			
	**Total**	**high**	**low**	**Pvalue**	**Total**	**high**	**low**	**Pvalue**
Age								
<=65	52(65%)	24(60%)	28(70%)	0.4819	46(58.23%)	26(63.41%)	20(52.63%)	0.4577
>65	28(35%)	16(40%)	12(30%)		33(41.77%)	15(36.59%)	18(47.37%)	

Gender								
FEMALE	15(18.75%)	8(20%)	7(17.5%)	1	8(10.13%)	3(7.32%)	5(13.16%)	0.6265
MALE	65(81.25%)	32(80%)	33(82.5%)		71(89.87%)	38(92.68%)	33(86.84%)	
Grade								
G1-2	42(52.5%)	15(37.5%)	27(67.5%)	**0.036**	39(49.37%)	16(39.02%)	23(60.53%)	0.0859
G3	19(23.75%)	13(32.5%)	6(15%)		24(30.38%)	16(39.02%)	8(21.05%)	
Stage								
Stage I-II	43(53.75%)	17(42.5%)	26(65%)	0.0901	41(51.9%)	15(36.59%)	26(68.42%)	**0.0034**
Stage III-IV	25(31.25%)	16(40%)	9(22.5%)		31(39.24%)	23(56.1%)	8(21.05%)	
T								
T1-2	39(48.75%)	17(42.5%)	22(55%)	0.6694	25(31.65%)	9(21.95%)	16(42.11%)	0.0566
T3-4	31(38.75%)	16(40%)	15(37.5%)		48(60.76%)	30(73.17%)	18(47.37%)	
M								
M0	58(72.5%)	26(65%)	32(80%)	0.414	61(77.22%)	32(78.05%)	29(76.32%)	0.3073
M1	2(2.5%)	2(5%)	0(0%)		6(7.59%)	5(12.2%)	1(2.63%)	
N								
N0	33(41.25%)	10(25%)	23(57.5%)	**0.0081**	32(40.51%)	11(26.83%)	21(55.26%)	**0.0105**
N1-3	37(46.25%)	24(60%)	13(32.5%)		40(50.63%)	27(65.85%)	13(34.21%)	
smoking								
NO	26(32.5%)	11(27.5%)	15(37.5%)	0.7023	20(25.32%)	10(24.39%)	10(26.32%)	1
YES	46(57.5%)	23(57.5%)	23(57.5%)		50(63.29%)	24(58.54%)	26(68.42%)	
alcohol								
NO	20(25%)	12(30%)	8(20%)	0.5188	26(32.91%)	16(39.02%)	10(26.32%)	0.2978
YES	58(72.5%)	28(70%)	30(75%)		52(65.82%)	24(58.54%)	28(73.68%)	

Table S4. List of differentially expressed genes in the high- and low-risk groups

gene	lowMean	highMean	logFC	*P*Value	fdr
SPRR2E	247.55135	89.98092	-1.46004	0.00123	0.01492
DPP4	4.43778	10.07434	1.18278	0.00709	0.04202
SLC39A5	2.05642	4.77468	1.21527	0.00569	0.03645
SPRR2A	596.02135	189.90033	-1.65012	0.00190	0.01906
CSTA	268.56706	89.54083	-1.58466	0.00041	0.00784
RAET1E	2.61748	0.71634	-1.86945	0.00236	0.02182
GOLT1A	1.97545	4.03091	1.02892	0.00125	0.01499
RHCG	290.84044	84.86746	-1.77694	0.00786	0.04532
APOH	0.10307	11.84132	6.84401	0.00029	0.00646
IL1RN	129.27112	49.19164	-1.39391	0.00646	0.03951
SPRR2F	55.01638	21.39254	-1.36275	0.00416	0.03011
FDCSP	82.60116	14.65671	-2.49460	0.00753	0.04397
POU3F1	2.58932	1.28394	-1.01199	0.00141	0.01601
SERPINA1	41.06964	121.54472	1.56534	0.00408	0.02973
COL17A1	98.84054	48.19178	-1.03632	0.00002	0.00158
RGL3	0.93628	2.25017	1.26502	0.00022	0.00558

MAL	118.83208	23.11110	-2.36227	0.00475	0.03272
AL356867.1	2.01087	0.67511	-1.57464	0.00303	0.02507
NGFR	11.59369	4.96323	-1.22399	0.00046	0.00851
PRSS1	0.50997	2.03511	1.99662	0.00079	0.01164
SBSN	101.20015	43.27220	-1.22570	0.00401	0.02948
DCDC2	0.52427	2.22089	2.08276	0.00073	0.01121
ZNF185	52.12512	23.04015	-1.17783	0.00201	0.01972
ABCG2	0.76365	1.90272	1.31708	0.00470	0.03257
IVL	50.42643	20.38646	-1.30657	0.00012	0.00398
KRT16	474.26587	231.67977	-1.03356	0.00143	0.01613
ALPI	0.65079	1.98986	1.61241	0.00374	0.02840
SALL4	0.92433	2.86470	1.63191	0.00000	0.00042
ELFN1-AS1	1.57378	4.71905	1.58426	0.00013	0.00424
SLC29A4	1.51369	4.13134	1.44854	0.00280	0.02398
EPGN	4.07065	1.36260	-1.57890	0.00545	0.03543
RNF157	1.00239	2.19743	1.13237	0.00015	0.00455
TMEM74B	0.96988	2.65836	1.45465	0.00001	0.00100
NCCRP1	48.05820	15.84109	-1.60111	0.00888	0.04872
AMBP	0.31460	4.85303	3.94729	0.00045	0.00847
TP53AIP1	3.18952	1.29052	-1.30539	0.00247	0.02252
CSTB	323.25097	142.32741	-1.18344	0.00215	0.02054
LYNX1	14.53727	6.70233	-1.11702	0.00626	0.03862
CERS3	9.67526	4.78655	-1.01531	0.00140	0.01593
SPINK7	18.04921	3.61355	-2.32045	0.00860	0.04794
BIRC7	0.17175	2.33174	3.76303	0.00861	0.04794
LINC00659	0.78692	1.85909	1.24030	0.00500	0.03369
MTATP6P2	0.77042	2.19853	1.51283	0.00141	0.01601
CYP2B6	0.84492	4.11461	2.28386	0.00066	0.01061
ECM1	75.18724	25.39043	-1.56620	0.00003	0.00195
CABYR	1.60871	4.02036	1.32142	0.00870	0.04809
CXCL5	3.10696	30.39492	3.29026	0.00653	0.03977
TM4SF5	8.79783	18.94283	1.10643	0.00377	0.02847
MIR378G	0.64994	1.46934	1.17680	0.00076	0.01138
FAM25A	24.24800	5.30375	-2.19278	0.00123	0.01491
LBP	0.07623	3.24363	5.41107	0.00251	0.02276
SYCP2	0.90584	2.30711	1.34876	0.00259	0.02309
LGALS7B	85.69522	22.74905	-1.91341	0.00160	0.01724
VIL1	8.10643	19.97625	1.30115	0.00557	0.03599
SYT13	3.62637	7.33049	1.01538	0.00095	0.01285
VGF	0.57704	2.50156	2.11610	0.00268	0.02348
G0S2	7.97166	17.10153	1.10117	0.00366	0.02799
S100A8	1343.34427	534.18317	-1.33042	0.00417	0.03011
LCE3A	3.73786	0.68417	-2.44979	0.00146	0.01632
LCE3D	40.99032	14.85315	-1.46451	0.00278	0.02392

ENDOU	3.92668	1.09587	-1.84123	0.00724	0.04266
CCL21	27.56871	10.05188	-1.45557	0.00140	0.01593
GDF15	12.13473	28.27722	1.22050	0.00025	0.00592
RAB3B	0.79435	2.98767	1.91118	0.00001	0.00121
A2ML1	51.32739	17.17288	-1.57960	0.00146	0.01632
MIR3646	0.52660	1.50715	1.51704	0.00088	0.01238
ANXA1	323.09753	145.94039	-1.14659	0.00350	0.02716
SPP1	64.72094	160.54137	1.31064	0.00192	0.01921
NKILA	0.79965	2.00018	1.32268	0.00724	0.04266
AC254629.1	0.75465	1.78689	1.24357	0.00117	0.01447
SATB2	0.82793	1.70876	1.04537	0.00000	0.00029
ABCC2	0.55168	2.16635	1.97337	0.00165	0.01752
RBP4	4.19154	11.80755	1.49416	0.00016	0.00458
PALM3	0.96435	2.44514	1.34229	0.00061	0.01009
STC2	1.95289	4.40010	1.17193	0.00023	0.00566
ALOX12	7.01889	2.90665	-1.27188	0.00015	0.00455
GRB14	0.86186	2.20065	1.35240	0.00331	0.02648
GBP6	52.50033	21.83319	-1.26580	0.00135	0.01565
TNNC2	0.65630	1.79608	1.45243	0.00006	0.00282
KRT6C	238.50721	79.15887	-1.59121	0.00401	0.02948
HSPA6	6.23444	17.09819	1.45551	0.00552	0.03575
C5orf66-AS1	5.49423	2.28799	-1.26384	0.00713	0.04222
KLK5	21.18096	7.16203	-1.56433	0.00078	0.01144
SPRR2C	37.60765	13.03011	-1.52918	0.00237	0.02192
KRT31	2.44978	0.84385	-1.53760	0.00173	0.01786
CRCT1	132.38657	26.73540	-2.30793	0.00041	0.00790
AL121772.1	0.69885	1.46649	1.06931	0.00110	0.01400
AC010547.2	0.59185	1.49295	1.33487	0.00272	0.02366
KRT16P4	1.91180	0.89106	-1.10134	0.00125	0.01501
TMPRSS11F	3.38370	1.40050	-1.27266	0.00503	0.03379
PPP1R3C	7.72025	2.64823	-1.54362	0.00563	0.03623
GUCA2A	0.33905	2.36815	2.80421	0.00635	0.03910
CRNN	224.12416	56.04269	-1.99970	0.00423	0.03040
EID3	0.74064	1.61337	1.12323	0.00006	0.00269
CRABP2	108.20455	40.00149	-1.43564	0.00072	0.01108
TMPRSS11D	25.28141	10.16731	-1.31414	0.00313	0.02555
MLXIPL	1.67053	5.05314	1.59687	0.00005	0.00237
FGFR4	6.63410	13.30829	1.00435	0.00017	0.00490
HOPX	11.49376	4.45570	-1.36713	0.00051	0.00917
WFDC12	3.13129	0.97009	-1.69057	0.00720	0.04253
APOA2	0.05993	10.84575	7.49973	0.00021	0.00547
LINC00888	1.20196	2.97106	1.30559	0.00000	0.00006
GSTA2	0.33587	1.95060	2.53796	0.00223	0.02102
HAS3	23.28801	11.03174	-1.07793	0.00075	0.01130

MIR559	1.68294	3.98311	1.24291	0.00006	0.00282
CNFN	434.73617	125.24195	-1.79542	0.00096	0.01296
KPRP	3.25137	0.68194	-2.25334	0.00052	0.00923
SLC7A2	1.97557	4.47873	1.18082	0.00460	0.03211
SPRR1A	1146.54047	385.24270	-1.57345	0.00038	0.00744
LYPD8	0.45117	3.64126	3.01269	0.00204	0.01996
SLURP1	41.25597	12.05015	-1.77555	0.00592	0.03742
SPRR1B	1794.60952	565.77974	-1.66536	0.00031	0.00662
CLDN9	0.55009	1.63707	1.57337	0.00053	0.00931
PRSS2	3.72218	11.73548	1.65666	0.00156	0.01700
UPK3B	5.58159	2.51129	-1.15224	0.00346	0.02705
HSPB8	21.05419	8.59251	-1.29296	0.00127	0.01510
CA8	1.16920	2.75079	1.23433	0.00762	0.04424
MTND2P28	129.84061	261.30775	1.00901	0.00009	0.00350
LYPD3	134.85609	66.29780	-1.02439	0.00496	0.03349
CPA2	0.38811	4.08494	3.39576	0.00235	0.02182
CLIC3	38.11419	12.76354	-1.57830	0.00220	0.02083
ZNF812P	8.40781	4.07702	-1.04422	0.00480	0.03290
TENT5B	13.38220	5.14378	-1.37941	0.00081	0.01171
KRT6B	481.21193	230.08341	-1.06452	0.00340	0.02682
TGM1	62.53611	18.63975	-1.74631	0.00606	0.03794
CSPG4	18.68639	8.71885	-1.09978	0.00201	0.01972
AL117382.1	0.79024	1.88514	1.25430	0.00356	0.02750
PPL	98.44690	40.10842	-1.29544	0.00271	0.02356
AC012354.2	0.71478	1.69689	1.24732	0.00169	0.01774
SLC2A3	4.40137	8.94300	1.02281	0.00130	0.01530
LCE3E	15.52242	5.98516	-1.37489	0.00106	0.01374
RNU7-143P	0.56810	1.64707	1.53570	0.00002	0.00158
RGMA	4.95618	2.21218	-1.16376	0.00054	0.00935
PLA2G3	1.78008	0.87017	-1.03258	0.00119	0.01459
MIR3189	0.81657	2.63594	1.69067	0.00132	0.01545
RNF225	3.06439	1.28572	-1.25302	0.00310	0.02541
LYPD5	4.40687	2.13067	-1.04844	0.00067	0.01066
CLDN6	0.28349	6.53926	4.52775	0.00183	0.01864
SPRR2D	188.17903	76.41805	-1.30012	0.00090	0.01255
DEFA6	3.37869	11.91114	1.81778	0.00007	0.00313
PDZK1	0.36864	1.82300	2.30601	0.00600	0.03763
STK31	0.78451	2.20533	1.49114	0.00005	0.00237
C2CD4A	2.53195	5.91207	1.22341	0.00897	0.04898
SLC26A3	1.25873	6.02604	2.25925	0.00754	0.04397
SPRR3	877.60948	235.29890	-1.89908	0.00557	0.03599
CXCL8	23.89552	56.17169	1.23310	0.00827	0.04674

Table S5. List of potential compounds and drugs in cMAP

rank	cmap name	mean	n	enrichment	*P*	specificity	percent non-null
1	alprostadil	-0.352	7	-0.784	0.00002	0.008	57
2	protoveratrine A	0.297	4	0.825	0.00147	0	50
3	pyrithyldione	-0.345	4	-0.828	0.00165	0.007	50
4	trichlormethiazide	0.465	4	0.817	0.00199	0	75
5	crotamiton	0.512	4	0.807	0.00257	0	75
6	ethambutol	0.496	5	0.709	0.00495	0	80
7	5230742	0.698	2	0.945	0.00567	0.0078	100
8	tocainide	-0.367	4	-0.752	0.00768	0.0387	50
9	pridinol	0.322	4	0.745	0.008	0	50
10	0317956-0000	-0.325	8	-0.536	0.01101	0.0678	50
11	vancomycin	-0.321	4	-0.719	0.01255	0.0552	50
12	mefexamide	-0.271	4	-0.709	0.01484	0.0134	50
13	aminophylline	0.35	4	0.706	0.01556	0.0323	50
14	5182598	0.62	2	0.908	0.01769	0.1373	100
15	syrosingopine	-0.526	4	-0.694	0.01846	0.0722	75
16	triprolidine	0.417	4	0.689	0.0195	0.0131	50
17	proxyphylline	0.501	4	0.681	0.02216	0	75
18	ticlopidine	0.359	5	0.62	0.02341	0.018	60
19	metrizamide	-0.451	4	-0.677	0.02399	0.0482	75
20	harmol	0.454	4	0.666	0.02773	0.0543	75
21	perphenazine	-0.352	5	-0.597	0.0305	0.1017	60
22	meropenem	0.333	4	0.657	0.03209	0.0244	50
23	antazoline	0.497	4	0.655	0.03312	0.0327	75
24	flunarizine	0.346	4	0.654	0.0333	0.0544	50
25	5155877	0.414	4	0.648	0.03654	0.1563	75
26	securinine	0.508	4	0.644	0.03863	0.1688	75
27	econazole	0.511	4	0.643	0.03923	0.1897	75
28	adenosine phosphate	-0.288	4	-0.633	0.04434	0.0331	50

Table S6. Targets of perphenazine

Pharma Model	Num Feature	Norm Fit	Symbol
1ggz_A_cavity_1	3	0.8752	CALML3
1zbu_C_cavity_2	5	0.5974	ERI1
2dgu_A_cavity_2	5	0.5903	SYNCRIP
1ufi_C_cavity_1	5	0.5819	CENPB
2elb_A_cavity_2	12	0.5807	APPL1
1so0_C_cavity_2	5	0.5756	GALM
1nu9_C_cavity_1	5	0.559	F2

3b7k_A_cavity_1	9	0.5459	ACOT12
2d86_A_cavity_2	5	0.5268	VAV3
2jis_A_cavity_3	5	0.5251	CSAD
1p32_C_cavity_1	6	0.4997	C1QBP
1eyb_A_cavity_2	6	0.4961	HGD
1h6e_A_cavity_1	6	0.4809	AP2M1
2z5f_A_cavity_1	6	0.4807	SULT1B1
2f3i_A_cavity_1	6	0.4787	POLR2H
3bhy_A_cavity_2	16	0.4763	DAPK3
3c5k_A_cavity_1	7	0.4745	HDAC6
2w4o_A_cavity_1	6	0.4742	CAMK4
1s9c_F_cavity_3	6	0.4716	HSD17B4
3gg6_A_cavity_1	6	0.47	NUDT18
1x86_A_cavity_2	6	0.4682	ARHGEF12
2d8h_A_cavity_1	6	0.4675	SH3YL1
1x9n_A_cavity_5	6	0.4666	LIG1
3dw8_B_cavity_2	15	0.4652	PPP2R1A
1yae_E_cavity_1	17	0.4621	GRIK1
2i6a_D_cavity_1	6	0.4616	ADK
1w98_B_cavity_1	6	0.4602	CCNE1
1n46_B_cavity_1	6	0.4595	THRB
2zfh_B_cavity_1	8	0.4582	CUTA
1htr_P_cavity_1	6	0.4564	PGC
2z7x_B_cavity_1	6	0.4467	TLR1
1wh0_A_cavity_1	6	0.4414	USP19
3bm4_A_cavity_1	6	0.4396	NUDT5
1n8s_A_cavity_2	6	0.439	PNLIP
2ozb_B_cavity_2	6	0.4382	SNU13
1x5n_A_cavity_1	6	0.4363	USH1C
1yhn_B_cavity_1	6	0.4361	RAB7A
2hzp_A_cavity_1	9	0.4276	KYNU
2bsk_D_cavity_1	8	0.4268	TIMM9
2rfj_B_cavity_1	7	0.4255	BRDT
2dmd_A_cavity_2	7	0.4253	ZFP64
2p2c_P_cavity_1	7	0.4249	CASP2
2qp4_A_cavity_2	7	0.4239	SULT2A1
2a98_A_cavity_2	8	0.4231	ITPKC
1nst_A_cavity_1	7	0.4212	NDST1
1w3b_B_cavity_1	7	0.4211	OGT
2daf_A_cavity_2	7	0.421	IQUB
2qlu_A_cavity_2	9	0.4207	ACVR2B
2dhg_A_cavity_1	7	0.4198	TRNAU1AP
3b68_A_cavity_1	7	0.4188	AR
1onq_C_cavity_1	7	0.4187	CD1A

1us1_B_cavity_5	7	0.418	AOC3
2r3v_D_cavity_1	7	0.4173	MVK
1jcn_A_cavity_1	7	0.4167	IMPDH1
1wjp_A_cavity_1	7	0.4164	ZBTB21
3g5k_C_cavity_1	9	0.4153	PDF
1xdt_T_cavity_3	7	0.4148	HBEGF
2zfu_B_cavity_1	7	0.4115	RRP8
1mj4_A_cavity_1	7	0.4111	SUOX
1x5f_A_cavity_1	8	0.4098	NEO1
2dlz_A_cavity_1	7	0.4097	VAV2
2ge9_A_cavity_2	7	0.4076	BTK
1ch4_A_cavity_1	9	0.4033	HBB
2aa7_A_cavity_1	7	0.4001	NR3C2
2he7_A_cavity_4	7	0.3996	EPB41L3
1v5w_B_cavity_1	7	0.3978	DMC1
2yqi_A_cavity_1	7	0.3977	HMGB3
1ef1_D_cavity_1	9	0.3974	MSN
1p4q_B_cavity_1	7	0.3903	CITED2
1wi3_A_cavity_2	7	0.39	SATB2
2uxw_A_cavity_1	15	0.3896	ACADVL
3fm0_A_cavity_1	9	0.3883	CIAO1
2vpi_A_cavity_1	7	0.3872	GMPS
1t9g_S_cavity_1	7	0.3866	ACADM
2vkq_A_cavity_2	15	0.3823	NT5C3A
1r6u_A_cavity_4	7	0.3776	WARS
1iau_A_cavity_1	7	0.3761	GZMB

Supplementary References

Forli S, Huey R, Pique ME, Sanner MF, Goodsell DS, Olson AJ. Computational protein-ligand docking and virtual drug screening with the AutoDock suite. *Nat Protoc*. 2016;11(5):905-919. doi:10.1038/nprot.2016.051

Trott O, Olson AJ. AutoDock Vina: improving the speed and accuracy of docking with a new scoring function, efficient optimization, and multithreading. *J Comput Chem*. 2010;31(2):455-461. doi:10.1002/jcc.21334

Guo C, Zeng F, Liu H, Wang J, Huang X, Luo J. Establish immune-related gene prognostic index for esophageal cancer. *Front Genet*. 2022;13:956915. doi: 10.3389/fgene.2022.956915.

Jiang SC, Tao SH, Chen SY, Xie H, Feng YJ. Characterization of pyroptosis-related genes in esophageal cancer and construction of a prognostic model. *Eur Rev Med Pharmacol Sci*. 2023 Jul;27(14):6592-6604. doi: 10.26355/eurrev_202307_33130.

Ren Q, Zhang P, Zhang X, Feng Y, Li L, Lin H, Yu Y. A fibroblast-associated signature predicts prognosis and immunotherapy in esophageal squamous cell cancer. *Front Immunol*. 2023;14:1199040. doi: 10.3389/fimmu.2023.1199040.

Xiong K, Tao Z, Zhang Z, Wang J, Zhang P. Identification and Validation of a

Prognostic Immune-Related Gene Signature in Esophageal Squamous Cell Carcinoma. *Front Bioeng Biotechnol*. 2022;10:850669. doi: 10.3389/fbioe.2022.850669.
